# Translation-independent association of mRNAs that encode protomers of the 5-HT_2A_-mGlu2 receptor complex

**DOI:** 10.1016/j.jbc.2025.110427

**Published:** 2025-06-26

**Authors:** Somdatta Saha, Javier González-Maeso

**Affiliations:** Department of Physiology and Biophysics, Virginia Commonwealth University School of Medicine, Richmond, Virginia, USA

**Keywords:** G protein-coupled receptors (GPCRs), GPCR heteromerization, co-translation, RNA-binding protein (RBP), transcript association

## Abstract

G protein-coupled receptors (GPCRs) constitute the largest family of plasma membrane proteins and regulate cell signaling by activating heterotrimeric G proteins. The serotonin 5-HT_2A_ receptor (5-HT_2A_R) and the metabotropic glutamate 2 receptor (mGluR2) are GPCRs that play a pivotal role in processes related to perception, memory, and mood regulation. These receptors can interact to form heteromeric GPCR complexes through direct physical interactions, which modulate the signaling and trafficking properties of both protomers. Co-translational association of mRNAs encoding subunits of heteromeric ion channels has been reported, but whether complex assembly of GPCRs occurs during translation remains unknown. Here, our *in vitro* data reveals evidence of co-translational modulation in *5-HT*_*2A*_*R* and *mGluR2* mRNAs following siRNA-mediated knockdown. Interestingly, immunoprecipitation of either 5-HT_2A_R or mGluR2, using an antibody targeting epitope tags at their N-terminus, results in detection of both transcripts associated with ribonucleoprotein complexes containing RPS24. Additionally, we demonstrate that the mRNA transcripts of *5-HT*_*2A*_*R* and *mGluR2* associate autonomously of their respective encoded proteins. Validation of this translation-independent association is extended *ex vivo* using mouse frontal cortex samples. Together, these findings provide mechanistic insights into the co-translational assembly of GPCR heteromeric complexes in mammalian cells, unraveling regulatory processes governing protein–protein interactions and complex formation.

The majority of proteins across all domains of life function as part of multimeric complexes (1, 2). G protein-coupled receptors (GPCRs) represent a highly diverse group of transmembrane proteins that exert various physiological effects (3, 4). This superfamily of membrane proteins can be grouped into classes A-F based on their primary sequence and signaling properties (5, 6). Molecular and functional studies indicate that class C GPCRs such as GABA_B_ receptors and metabotropic glutamate receptors (mGluRs) behave as obligate dimers (7, 8, 9). Additionally, while class A GPCRs were traditionally recognized for their efficient functioning as monomers; at least when reconstituted into a phospholipid bilayer (10, 11, 12), there is a growing body of evidence suggesting the potential for class A GPCRs to form homodimers/heterodimers and oligomers. These complexes exhibit distinct pharmacological, trafficking, and functional properties compared to their parent monomeric forms (13, 14, 15, 16). Although the physiological relevance of GPCR heteromerization is not fully established, it holds promise for generating novel drug target combinations and fine-tuning the structure and function of one or more GPCRs involved in the complex to enhance therapeutic strategies.

The serotonin (5-hydroxytryptamine, or 5-HT) 2A receptor (5-HT_2A_R) which belongs to class A, along with the class C mGluR2, are GPCRs involved in processes related to cognition, perception, and mood regulation. They constitute significant targets for various psychoactive substances, including psychedelics (also known as classical hallucinogens) such as psilocybin or lysergic acid diethylamide (LSD), as well as antipsychotics such as clozapine or risperidone (17, 18, 19, 20, 21, 22, 23). Several lines of evidence suggest that these two neurotransmitter GPCRs can physically interact: influencing G protein coupling, function, and trafficking (24, 25, 26, 27, 28). Insight into the role of this 5-HT_2A_R-mGluR2 complex in agonist-induced endocytic processes has been provided (29), yet much remains unknown about the assembly of their component protomers in terms of location, timing, and mechanism. Understanding this is crucial, as protein complexes serve as fundamental organizational units in the proteome, and their assembly within the crowded cellular environment is a complex task (30).

Cells employ various strategies to ensure faithful and efficient assembly, ranging from random subunit collisions (31, 32) to the involvement of cellular chaperones (33, 34) and dedicated assembly organelles (35). Another mechanism involves immediate co-translational folding and the simultaneous association of binding partners, preventing premature or unintended interactions of nascent peptides. While early biochemical evidence in prokaryotes demonstrated co-translational enzymatic activity in nascent multimeric enzymes (36), this concept has been extended to eukaryotic heteromeric complexes (37, 38, 39, 40). For example, it was established that alternative mRNA transcripts encoding *hERG1a* and *hERG1b* subunits, which assemble to produce the cardiac K_v_ channel (41), are physically associated during translation (42). However, to our knowledge, there is currently no biological evidence supporting the co-translational association of nascent GPCRs in mammalian cells.

Previous findings indicated a crosstalk mechanism between *5-HT*_*2A*_*R* and *mGluR2* affecting transcriptional processes. For instance, 5-HT_2A_R knockout mice exhibited reduced cortical expression of *mGluR2* mRNA (43), and chronic treatment with atypical antipsychotics downregulated cortical *mGluR2* mRNA *via* 5-HT_2A_R (44, 45). Building on these observations, here we explored the possibility of co-translational association of mRNA encoding protomers of the heteromeric 5-HT_2A_R-mGluR2 complex.

## Results

### Cross-regulation of mGluR2 mRNA and protein levels by 5-HT_2A_R

**Figure 1:**
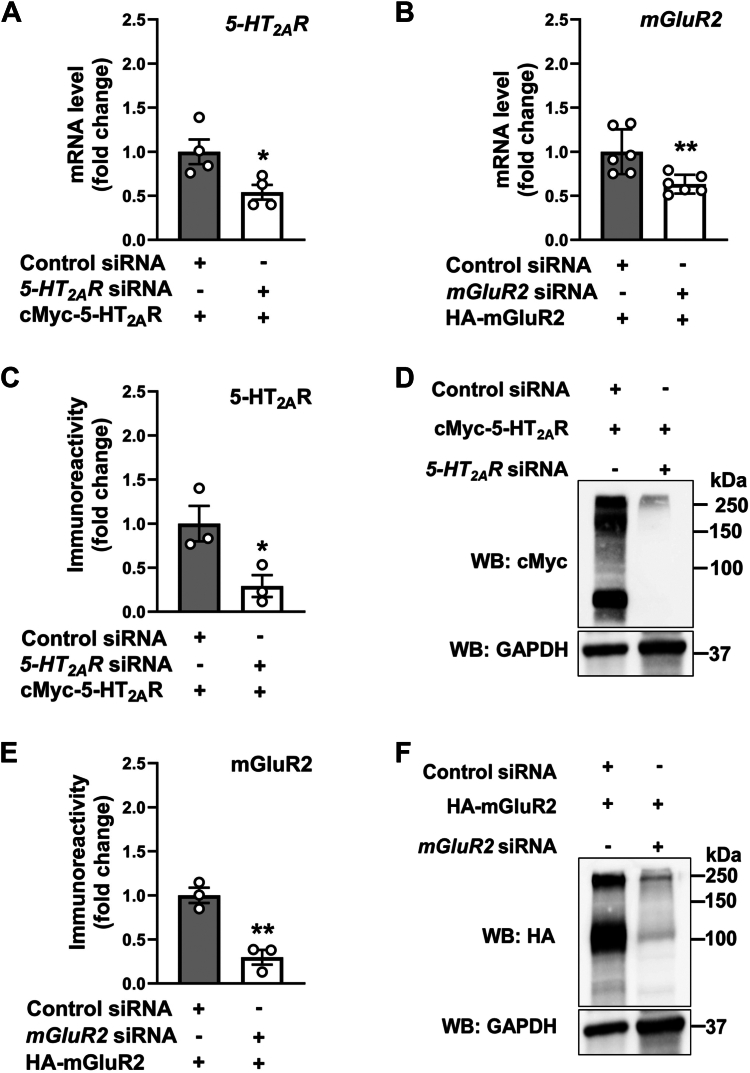
**siRNA-mediated knockdown of *5-HT*_*2A*_*R* and *mGluR2*.***A–F*, HEK293 cells were transfected with non-targeting siRNA, *5-HT*_*2A*_*R* siRNA (*A*, *C*, *D*) or mGluR2 siRNA (*B*, *E*, *F*). Forty-eight hours after siRNA transfection, cells were transfected with pcDNA3.1-cMyc-5-HT_2A_R (*A*, *C*, *D*) or pcDNA3.1-HA-mGluR2 (*B*, *E*, *F*). RNA and protein extractions were carried out 24 h following DNA transfection. Knockdown efficiency was assessed by RT-qPCR for *5-HT*_*2A*_*R* (*A*) and *mGluR2* (*B*) mRNAs (n = 4–6 independent samples), and by Western blot for 5-HT_2A_R (*C*) and mGluR2 (*E*) (n = 3 independent experiments). Representative immunoblots are shown (*D*, *F*). Unpaired two-tailed Student’s *t* test (∗*p* < 0.05, ∗∗*p* < 0.01). Data show mean ± s.e.m.

**Figure 2:**
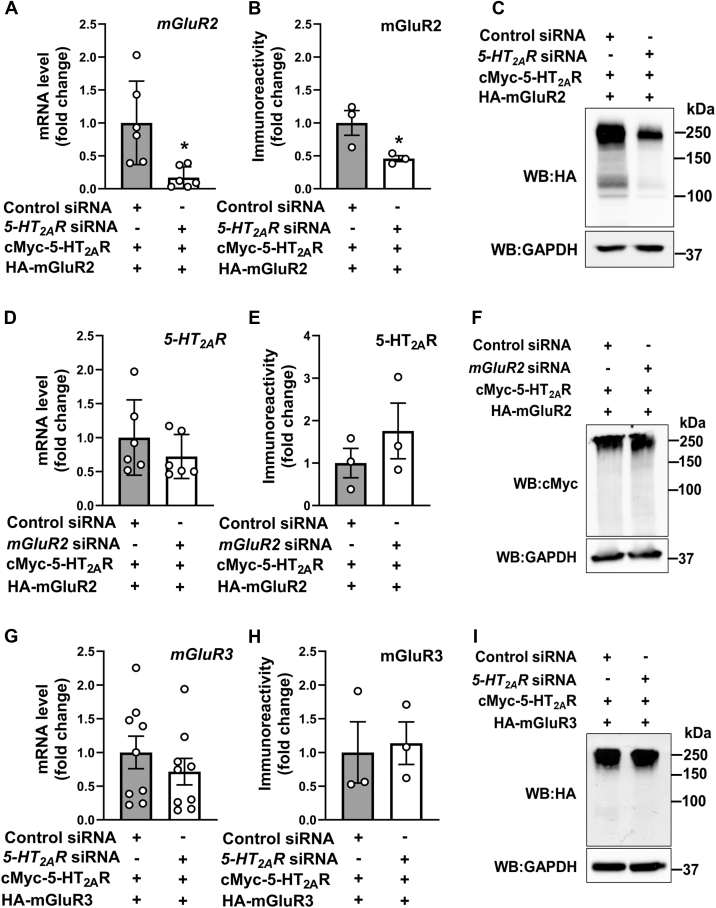
**Selective cross-regulation of *mGluR2* expression by 5-HT_2A_R.***A–C*, HEK293 cells were transfected with non-targeting siRNA or *5-HT*_*2A*_*R* siRNA. Forty-eight hours after siRNA transfection, cells were co-transfected with pcDNA3.1-cMyc-5-HT_2A_R and pcDNA3.1-HA-mGluR2. RNA and protein extractions were carried out 24 h following DNA transfection. *mGluR2* mRNA was assessed by RT-qPCR (n = 6 independent samples) (*A*), and mGluR2 immunoreactivity was assessed by Western blot (n = 3 independent experiments) (*B*). Representative immunoblots are shown (*C*). *D–F*, HEK293 cells were transfected with non-targeting siRNA or *mGluR2* siRNA. Forty-eight hours after siRNA transfection, cells were co-transfected with pcDNA3.1-cMyc-5-HT_2A_R and pcDNA3.1-HA-mGluR2. RNA and protein extractions were carried out 24 h following DNA transfection. *5-HT*_*2A*_*R* mRNA was assessed by RT-qPCR (n = 6 independent samples) (*D*), and 5-HT_2A_R immunoreactivity was assessed by Western blot (n = 3 independent experiments) (*E*). Representative immunoblots are shown (*F*). *G–I*, HEK293 cells were transfected with non-targeting siRNA or *5-HT*_*2A*_*R* siRNA. Forty-eight hours after siRNA transfection, cells were co-transfected with pcDNA3.1-cMyc-5-HT_2A_R and pcDNA3.1-HA-mGluR3. RNA and protein extractions were carried out 24 h following DNA transfection. *mGluR3* mRNA was assessed by RT-qPCR (n = 9 independent samples) (*G*), and mGluR3 immunoreactivity was assessed by Western blot (n = 3 independent experiments) (*H*). Representative immunoblots are shown (*I*). Unpaired two-tailed Student’s *t* test (∗*p* < 0.05). Data show mean ± s.e.m.

To investigate the reciprocal regulation of 5-HT_2A_R and mGluR2 at both the mRNA and the protein levels, we aimed to elucidate the impact of individually silencing these genes in a co-expression system. Initially, we assessed the influence of *5-HT*_*2A*_*R* small-interfering RNA (siRNA) and *mGluR2* siRNA in HEK293 cells transfected to co-express cMyc-5HT_2A_R and HA-mGluR2, respectively. Each siRNA, as compared to a non-targeting siRNA control, led to an approximately 50% reduction in the corresponding mRNA (Fig. 1, *A* and *B*), and immunoreactive levels (Fig. 1, *C*–*F*). Interestingly, silencing *5-HT*_*2A*_*R* with its specific siRNA in cells co-expressing 5-HT_2A_R and mGluR2 resulted in a significant decrease in *mGluR2* mRNA (Fig. 2*A*) and immunoreactive levels (Fig. 2, *B* and *C*), each reduced by almost half. However, downregulation of *mGluR2* by its specific siRNA in cells co-expressing both receptors did not significantly impact the mRNA (Fig. 2*D*) and immunoreactive levels (Fig. 2, *E* and *F*) of 5-HT_2A_R. To confirm the specificity of this cross-regulation between 5-HT_2A_R and mGluR2, we examined the expression of mGluR3 (mRNA and protein) upon downregulation of *5-HT*_*2A*_*R* in cells co-expressing cMyc-5HT_2A_R and HA-mGluR3. The expression of *mGluR3* mRNA (Fig. 2*G*) and immunoreactivity levels (Fig. 2, *H* and *I*) remained unchanged following *5-HT*_*2A*_*R* silencing. This selective effect on *mGluR2* was further validated using a second, independent siRNA targeting a different region of the *5-HT*_*2A*_*R* gene (Fig. S1).

Thus, the simultaneous decrease in *mGluR2* mRNA upon silencing of *5-HT*_*2A*_*R* mRNA suggests a potential physical association between the co-expressed transcripts. The concurrent reduction in their protein levels upon downregulation of 5-HT_2A_R further supports the idea of transcriptional regulation during translation of the interacting proteins.

### Co-translational assembly of 5-HT_2A_R and mGluR2 transcripts

**Figure 3:**
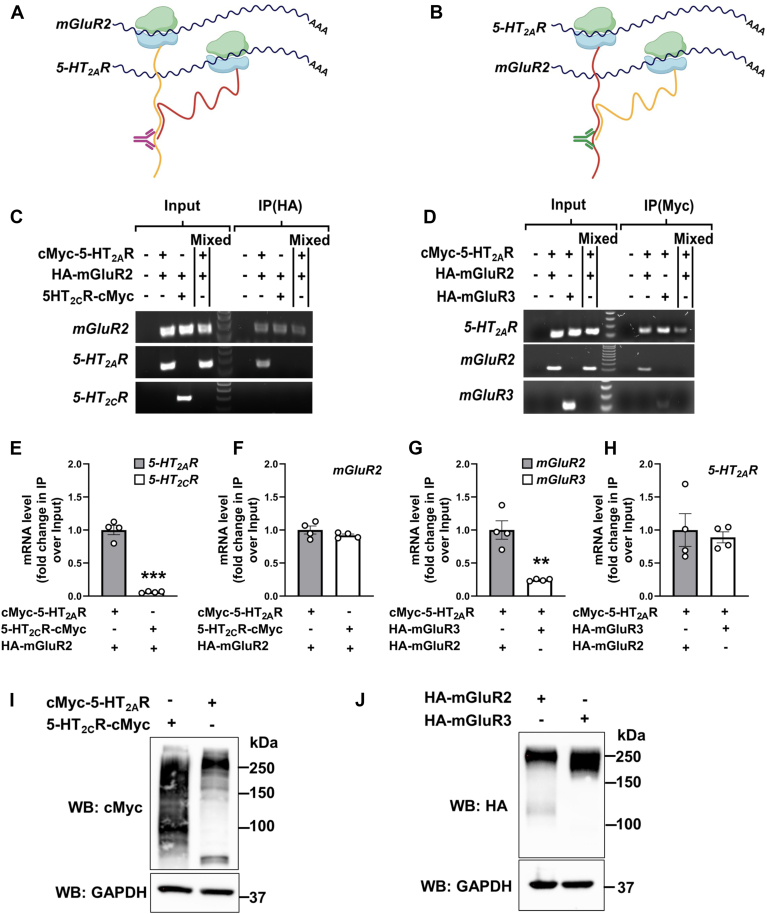
**Co-translational assembly of *5-HT*_*2A*_*R* and *mGluR2* mRNAs.***A*, schematic representation illustrating the co-translational association of 5-HT_2A_R and mGluR2 polypeptides, depicted in *red* and *yellow*, respectively, as they emerge from ribosomes shown in *blue* and *green*. The polypeptides originate from neighboring *5-HT*_*2A*_*R* and *mGluR2* transcripts, depicted in *black*. The mGluR2 targeting antibody (anti-HA, *magenta*) used for immunoprecipitation of the mRNA/protein complex is indicated, bound to the N-terminal of mGluR2. *B*, schematic representation illustrating the co-translational association of 5-HT_2A_R and mGluR2 polypeptides, depicted in *red* and *yellow*, respectively, as they emerge from ribosomes shown in *blue* and *green*. The polypeptides originate from neighboring *5-HT*_*2A*_*R* and *mGluR2* transcripts, depicted in *black*. The 5-HT_2A_R targeting antibody (anti-cMyc, *dark green*) used for immunoprecipitation of the mRNA/protein complex is indicated, bound to the N-terminal of 5-HT_2A_R. *C*, HEK293 cells were co-transfected with pcDNA3.1-HA-mGluR2, and pcDNA3.1-cMyc-5-HT_2A_R or pcDNA3.1-5-HT_2C_R-cMyc constructs, or mock. Images show representative RT-PCR products for *mGluR2*, *5-HT*_*2A*_*R* and *5-HT*_*2C*_*R* transcripts from HEK293 cells before (Input) and after immunoprecipitation (IP) using an anti-HA antibody. For control, cells separately expressing the c-Myc- or HA-tagged forms were mixed. Data are representative from three independent experiments. *D*, HEK293 cells were co-transfected with pcDNA3.1-cMyc-5-HT_2A_R, and pcDNA3.1-HA-mGluR2 or pcDNA3.1-HA-mGluR3 constructs, or mock. Images show representative RT-PCR products for *5-HT*_*2A*_*R*, *mGluR2* and *mGluR3* transcripts from HEK293 cells before (Input) and after immunoprecipitation (IP) using an anti-cMyc antibody. For control, cells separately expressing the c-Myc- or HA-tagged forms were mixed. Data are representative of three independent experiments. *E* and *F*, HEK293 cells were co-transfected with pcDNA3.1-HA-mGluR2, and pcDNA3.1-cMyc-5-HT_2A_R or pcDNA3.1-5-HT_2C_R-cMyc constructs. Subsequently, RNP complexes isolated through RIP assays employing an anti-HA antibody underwent processing for RNA isolation and RT-qPCR assays for the detection of *5-HT*_*2A*_*R* and *5-HT*_*2C*_*R* (*E*), and *mGluR2* (*F*) transcripts. Data are shown as fold change of IP/input (n = 4 independent samples) (*E* and *F*). *G* and *H*, HEK293 cells were co-transfected with pcDNA3.1-cMyc-5-HT_2A_R, and pcDNA3.1-HA-mGluR2 or pcDNA3.1-HA-mGluR3 constructs. Subsequently, RNP complexes isolated through RIP assays employing an anti-cMyc antibody underwent processing for RNA isolation and RT-qPCR assays for the detection of *mGluR2* and *mGluR3* (*G*), and *5-HT*_*2A*_*R* (*H*) transcripts. Data are shown as fold change of IP/input (n = 4 independent samples) (*G* and *H*). *I*, immunoblot with an anti-cMyc antibody in HEK293 cells transiently transfected with pcDNA3.1-c-Myc-5-HT_2A_R-mCherry or pcDNA3.1-5-HT_2C_R-cMyc-mCitrine. *J*, immunoblot with an anti-HA antibody in HEK293 cells transiently transfected with pcDNA3.1-HA-mGluR2-mCitrine or pcDNA3.1-HA-mGluR3-mCitrine. Unpaired two-tailed Student’s *t* test (*E*–*H*) (∗∗*p* < 0.01, ∗∗∗*p* < 0.001). Data show mean ± s.e.m.

We thus posited that if *5-HT*_*2A*_*R* and *mGluR2* transcripts physically associated during translation, it would be feasible to immunoprecipitate both transcripts using an antibody against the N-terminus of either of their nascent proteins. To assess this, HEK293 cells underwent co-transfection with cMyc-5-HT_2A_R and HA-mGluR2. Subsequently, ribonucleoprotein (RNP) complexes were extracted using a conventional RNP immunoprecipitation (RIP) assay, employing an anti-HA antibody for the immunoprecipitation of the nascent HA-mGluR2 polypeptide (for validation of the cellular fractionation protocol using markers of cellular compartments, including cytoplasmic α-Tubulin and nuclear Lamin A/C, see Fig. S2, *A* and *B*). This was followed by RNA isolation and reverse transcription PCR (RT-PCR) assays to identify transcripts associated with the mGluR2 protein (Fig. 3*A*). Likewise, an anti-cMyc antibody was used to immunoprecipitate the nascent cMyc-5-HT_2A_R protein, and RNA isolation and RT-PCR assays were performed to identify transcripts linked with the 5-HT_2A_R protein (Fig. 3*B*). Notably, our findings indicate that the anti-HA antibody immunoprecipitates not just the mGluR2 protein (Fig. S3*A*) but also the corresponding mRNAs for *mGluR2* and *5-HT*_*2A*_*R* (Fig. 3*C*). Similarly, the anti-cMyc antibody was capable of immunoprecipitating not only the 5-HT_2A_R protein (Fig. S3*B*) but also the associated *5-HT*_*2A*_*R* and *mGluR2* mRNAs (Fig. 3*D*). Importantly, this association was significantly reduced in cells co-expressing 5-HT_2C_R-cMyc and HA-mGluR2 (Fig. 3*C*), or cMyc-5-HT_2A_R and HA-mGluR3 (Fig. 3*D*), and was absent in cells individually transfected with cMyc-5-HT_2A_R or HA-mGluR2 and mixed post-transfection (Fig. 3, *C* and *D*). These results were further confirmed by RIP followed by reverse transcription quantitative PCR (RT-qPCR) (Fig. 3, *E*–*H*). Immunoblot analyses confirmed expression of the tagged constructs at their expected molecular weights (Fig. 3, *I* and *J*), and the specificity of the RIP assays was validated using non-immune immunoglobulin (IgG) as a negative control (Fig. S4).

**Figure 4:**
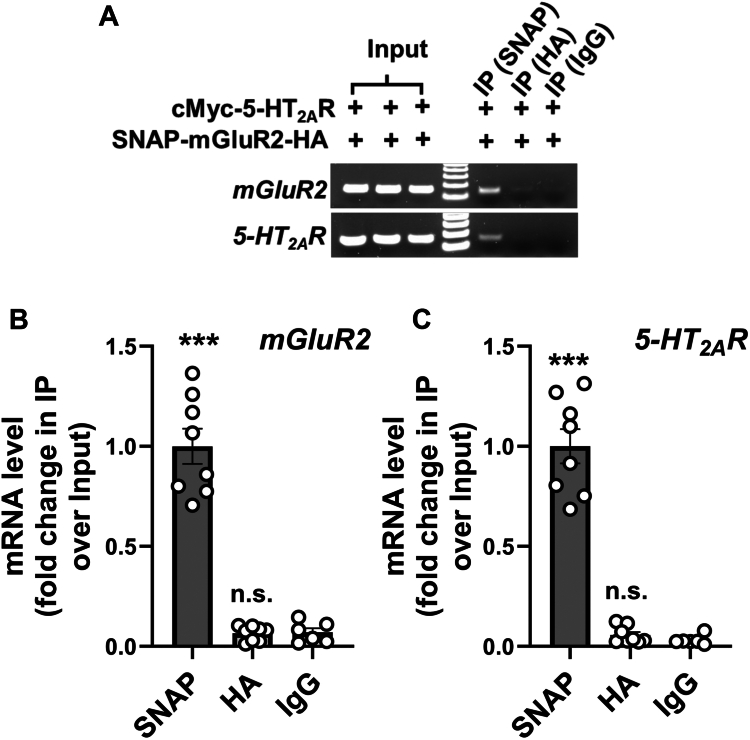
**Selective immunoprecipitation of *GPCR* transcripts in RIP assays using C- and N-terminal antibodies.***A*–*C*, HEK293 cells were co-transfected with pcDNA3.1-cMyc-5-HT_2A_R and pcDNA3.1-SNAP-mGluR2-HA constructs. Images show representative RT-PCR products for *5-HT*_*2A*_*R* and *mGluR2* transcripts from HEK293 cells before (Input) and after immunoprecipitation (IP) using either anti-SNAP or anti-HA antibodies, or non-immune immunoglobulin (IgG) as a control. Data are representative from three independent experiments (*A*). Quantification of *mGluR2* (*B*) and *5-HT*_*2A*_*R* (*C*) mRNA levels (IP/input relative to SNAP) by RT-qPCR analysis (n = 6–8 independent samples) (*B* and *C*). One-way ANOVA followed by Bonferroni’s *post hoc* test (*B* and *C*) (∗∗∗*p* < 0.001; ns, not significant). Data show mean ± s.e.m.

As an additional internal control, we evaluated the outcome of RIP assays using an antibody targeting the C-terminus of one of the two GPCRs, ensuring it recognizes only the fully translated protein. Similar to our previous assays, HEK293 cells were co-transfected with cMyc-5-HT_2A_R and SNAP-mGluR2-HA. As expected, immunoprecipitation assays with an anti-HA antibody targeting the C-terminus of SNAP-mGluR2-HA did not detect the *mGluR2* transcript, whereas it was successfully detected with an anti-SNAP antibody targeting the N-terminus—both in comparison to RIP assays using IgG as a negative control (Fig. 4*A*). Additionally, the anti-SNAP antibody immunoprecipitated the *5-HT*_*2A*_*R* transcript, an effect not observed with the HA-antibody (Fig. 4*A*). These findings were further corroborated through RIP assays followed by RT-qPCR (Fig. 4, *B* and *C*).

### Association of 5-HT_2A_R and mGluR2 transcripts is independent of protein translation

**Figure 5:**
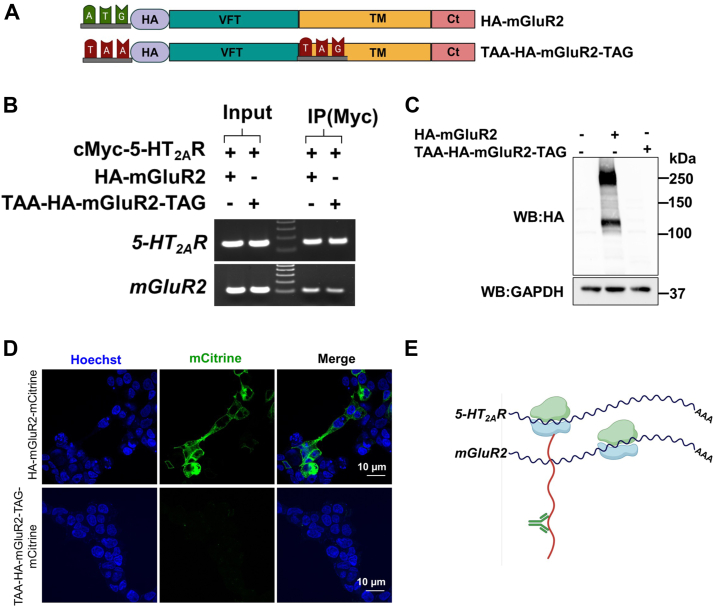
**Translation-independent association of mRNAs encoding *5-HT*_*2A*_*R* and *mGluR2*.***A*, schematic representation of the mGluR2 cDNA constructs, including “wild-type” HA-mGluR2, and TAA-HA-mGluR2-TAG which contains two stop codons at the start codon and at position Ala681 (located in the intracellular half of transmembrane four). *B*, HEK293 cells were co-transfected with pcDNA3.1-cMyc-5-HT_2A_R, and pcDNA3.1-HA-mGluR2 or pcDNA3.1-TAA-HA-mGluR2-TAG constructs. Images show representative RT-PCR products for *5-HT*_*2A*_*R* and *mGluR2* transcripts from HEK293 cells before (Input) and after immunoprecipitation (IP) using an anti-cMyc antibody. Data are representative from three independent experiments. *C*, immunoblot demonstrating loss of HA-mGluR2 protein in HEK293 cells transfected with the TAA-HA-mGluR2-TAG construct, compared to “wild-type” HA-mGluR2 control. *D*, confocal micrographs of HEK293 cells transiently transfected with pcDNA3.1-HA-mGluR2-mCitrine or pcDNA3.1-TAA-HA-mGluR2-TAG-mCitrine (*green*). Nuclei were stained with Hoechst (*blue*). Scale bars, 10 μM. *E*, schematic showing anti-cMyc antibody used to immunoprecipitate 5-HT_2A_R protein and association of *5-HT*_*2A*_*R* and *mGluR2* transcripts in the absence of mGluR2 protein.

Next, to explore whether the interaction between transcripts relied solely on the interaction between their corresponding proteins, we impeded the translation of the HA-mGluR2 construct by substituting the start codon (ATG) and the codon for Ala681, located in the intracellular half of transmembrane domain four (26), with two distinct stop codons (TAA and TAG), respectively, in the mGluR2 cDNA (TAA-HA-mGluR2-TAG) (Fig. 5*A*). As expected, although the *mGluR2* gene could produce a stable transcript (Fig. 5*B*), it was unable to translate a functional protein compared to its wild-type counterpart (Figs. 5*C* and S5). Visualization of mCitrine also revealed that full-length translation occurs only in cells transfected with the “wild-type” HA-mGluR2-mCitrine construct, and not in those transfected with TAA-HA-mGluR2-TAG-mCitrine (Fig. 5*D*). Interestingly, in cells co-transfected with cMyc-5-HT_2A_R and TAA-HA-mGluR2-TAG, immunoprecipitation of 5-HT_2A_R by the anti-cMyc antibody still effectively pulled down both *5-HT*_*2A*_*R* and *TAA-HA-mGluR2-TAG* transcripts (Fig. 5*B*). Thus, the transcripts associate even when the nascent mGluR2 and 5-HT_2A_R polypeptides do not interact, suggesting that an interaction at the mRNA level integrates the transcripts that form the 5-HT_2A_R-mGluR2 heterocomplex (Fig. 5*E*).

**Figure 6:**
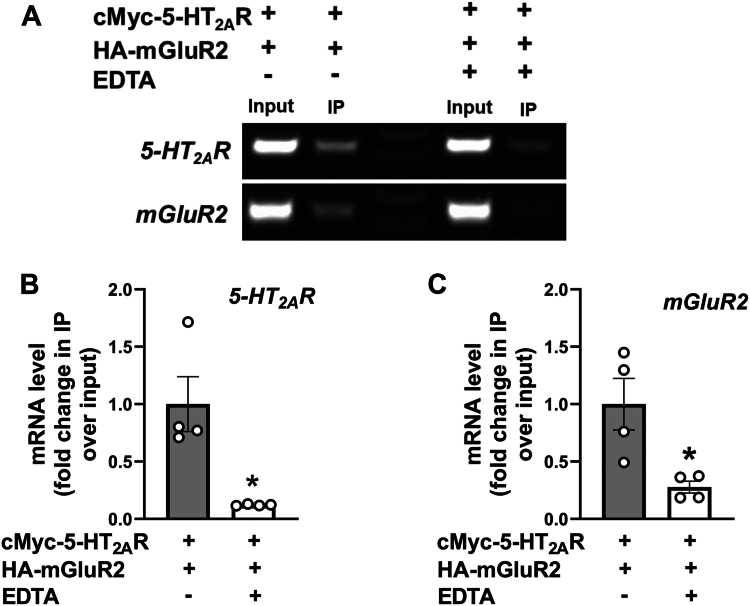
**Polysome dissociation by EDTA reduces mRNA association of *5-HT*_*2A*_*R* and *mGluR2*.***A*, HEK293 cells were co-transfected with pcDNA3.1-cMyc-5-HT_2A_R and pcDNA3.1-HA-mGluR2 constructs. Representative RT-PCR products showing *5-HT*_*2A*_*R* and *mGluR2* transcripts from HEK293 cells before (Input) and after immunoprecipitation (IP) with an anti-HA antibody. Data are representative from three independent experiments. *B* and *C*, HEK293 cells were co-transfected with pcDNA3.1-cMyc-5-HT_2A_R and pcDNA3.1-HA-mGluR2. Subsequently, RNP complexes isolated through RIP assays employing an anti-HA antibody underwent processing for RNA isolation and RT-qPCR assays for the detection of *5-HT*_*2A*_*R* (*B*) and *mGluR2* (*C*) transcripts. Data are shown as fold change of IP/input (n = 4 independent samples) (*B* and *C*). RIP assays were performed in the presence and absence of EDTA (25 mM) (*A*–*C*). Unpaired two-tailed Student’s *t* test (*B* and *C*) (∗*p* < 0.05). Data show mean ± s.e.m.

In addition, such translational-independent association between *5-HT*_*2A*_*R* and *mGluR2* was observed to decrease following EDTA treatment (Fig. 6*A*), which leads to polysome dissociation, resulting in free ribosomal subunits and mRNA (46). This effect of EDTA was further confirmed by RIP followed by RT-qPCR analysis (Fig. 6, *B* and *C*). Together, these findings suggest that, while there is no need for the two nascent peptides to interact to achieve the association of mRNAs encoding *5-HT*_*2A*_*R* and *mGluR2*, the EDTA-induced detachment of ribosomes from the mRNA strand also reduces the ability of the anti-HA antibody to pull down mRNA transcripts associated with the nascent HA-tagged protein partner.

### mRNA regions encoding extracellular and transmembrane domains of mGluR2 associated with the 5-HT_2A_R transcript

**Figure 7:**
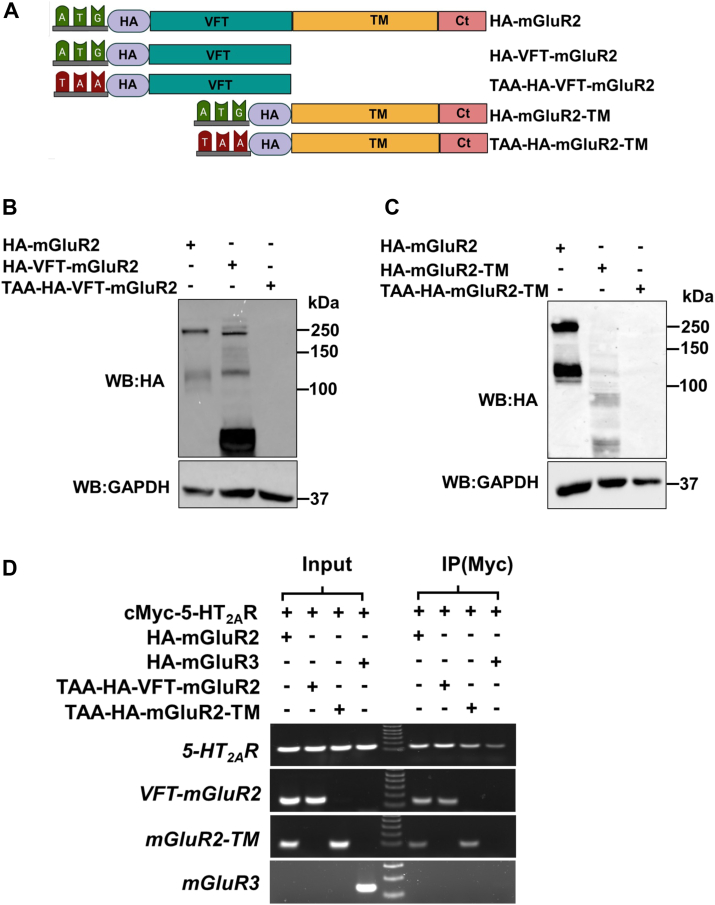
**Identification of *mGluR2* mRNA regions mediating association with *5-HT*_*2A*_*R* transcripts.***A*, schematic representation of the mGluR2 cDNA constructs, including “wild-type” HA-mGluR2, HA-VFT-mGluR2 which encodes the Venus flytrap domain, TAA-HA-VFT-mGluR2 which contains a stop codon at the start codon position, HA-mGluR2-TM which encodes the transmembrane domains, and TAA-HA-mGluR2-TM which contains a stop codon at the start codon position (VFT, Venus flytrap domain; TM, transmembrane domain; Ct, C-terminal tail). *B*, immunoblot with an anti-HA antibody in HEK293 cells transiently transfected with pcDNA3.1-HA-mGluR2, pcDNA3.1-HA-VFT-mGluR2, or pcDNA3.1-TAA-HA-VFT-mGluR2. *C*, immunoblot with an anti-HA antibody in HEK293 cells transiently transfected with pcDNA3.1-HA-mGluR2, pcDNA3.1-HA-mGluR2-TM, or pcDNA3.1-TAA-HA-mGluR2-TM. *D*, HEK293 cells were co-transfected with pcDNA3.1-cMyc-5-HT_2A_R, and pcDNA3.1-HA-mGluR2, pcDNA3.1-HA-mGluR3, pcDNA3.1-TAA-HA-VFT-mGluR2 or pcDNA3.1-TAA-HA-mGluR2-TM constructs. Images show representative RT-PCR products for *5-HT*_*2A*_*R*, *mGluR2*, *mGluR3*, *VFT-mGluR2*, and *mGluR2-TM* transcripts from HEK293 cells before (Input) and after immunoprecipitation (IP) using an anti-cMyc antibody. Data are representative from three independent experiments.

Class C GPCRs, including mGluR2, possess two main protein domains: the extracellularly located Venus flytrap (VFT) domain (which is responsible for both ligand binding and recognition) and the transmembrane domain (which passes through the cell membrane seven times) (7). To determine the mRNA region enabling the *mGluR2* transcript to associate with its *5-HT*_*2A*_*R* transcript counterpart within RNP complexes, we generated two HA-mGluR2 constructs encoding either the VFT domain (HA-VFT-mGluR2) or the transmembrane domain (HA-mGluR2-TM) (Fig. 7*A*). Transfection of HEK293 cells resulted in the detection of HA-tagged proteins by immunoblotting with an anti-HA antibody at their expected molecular weights (Fig. 7, *B* and *C*). However, this did not occur when the start codon (ATG) was replaced with a stop codon (TAA) in the TAA-HA-VFT-mGluR2 (Fig. 7, *A* and *B*) and TAA-HA-mGluR2-TM (Fig. 7, *A* and *C*) cDNA constructs. Interestingly, we found that an anti-cMyc antibody targeting the nascent cMyc-5-HT_2A_R effectively co-immunoprecipitated both the *TAA-HA-VFT-mGluR2* and *TAA-HA-mGluR2-TM* transcripts to a similar extent as that of the “wild-type” *HA-mGluR2*; compared to *HA-mGluR3*, which served as a negative control (Fig. 7*D*).

### Identification of RNA-binding proteins that enable co-translation of 5-HT_2A_R and mGluR2

**Figure 8:**
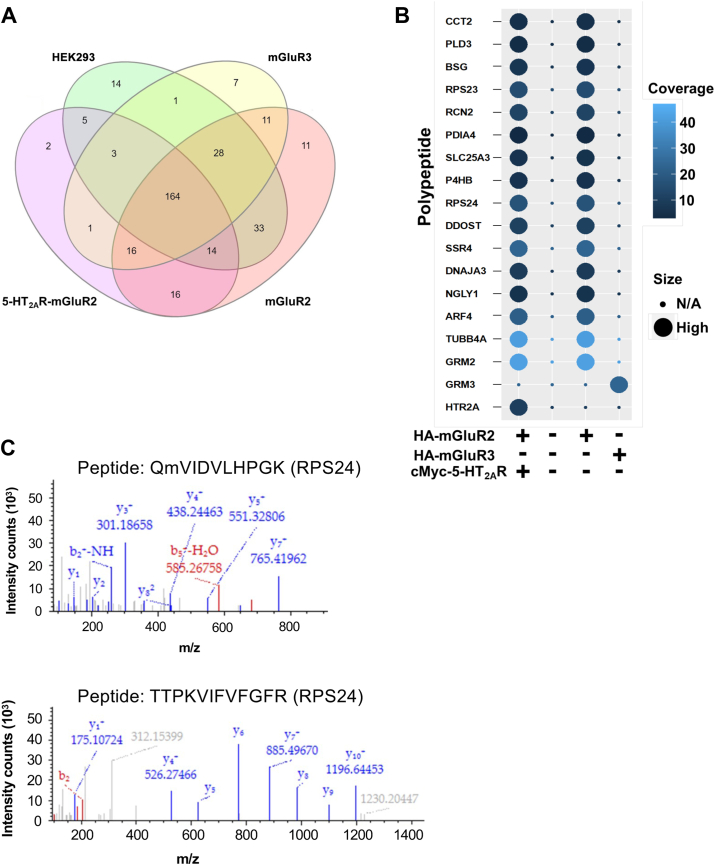
**Identification of RBPs associated with *5-HT*_*2A*_*R* and *mGluR2* co-translation.***A*, venn diagram showing the overlap of identified polypeptides by LC-MS/MS in parental HEK293 cells, cells transfected solely with pcDNA3.1-HA-mGluR2 or pcDNA3.1-HA-mGluR3, and cells co-transfected with pcDNA3.1-HA-mGluR2 and pcDNA3.1-cMyc-5HT_2A_R following RIP assay using the anti-HA antibody. *B*, dot plot depicting polypeptides present in HEK293 cells transfected solely with pcDNA3.1-HA-mGluR2 as well as in cells co-transfected with pcDNA3.1-HA-mGluR2 and pcDNA3.1-cMyc-5HT_2A_R, but not in parental HEK293 cells or cells transfected solely with pcDNA3.1-HA-mGluR3. *C*, LC-MS/MS analysis identifying with high confidence two tryptic peptides – QmVIDVLHPGK (*top*) and TTPKVIFVFGFR (*bottom*) – from RPS24. Mass-to-Charge (m/z) peptide fragments for the y ions (*blue*) and the b ions (*red*) are displayed in the spectra.

To explore the potential involvement of RNA-binding proteins (RBPs) in facilitating the co-translation of 5-HT_2A_R and mGluR2 proteins, we conducted mass spectrometry analysis on cells transfected solely with HA-mGluR2 or HA-mGluR3, along with those co-transfected with HA-mGluR2 and cMyc-5HT_2A_R, comparing them to parental HEK293 cells. As above, following the isolation of RNP complexes through a conventional RIP assay using an anti-HA antibody, the immunoprecipitated fractions underwent LC-MS/MS analysis. This analysis facilitated the identification of 326 polypeptides, which were found to be differentially abundant or absent throughout all four experimental conditions (Fig. 8*A*, and Table S1). Out of these, 16 polypeptides were found in cells that expressed HA-mGluR2 alone, and in cells that co-expressed cMyc-5-HT_2A_R and HA-mGluR2. However, these polypeptides were not detected in cells expressing HA-mGluR3 alone or in the parental HEK293 cells (Fig. 8, *A* and *B*). Interestingly, two of the polypeptides were identified as RBP – RPS23 and RBP – RPS24. Further bioinformatics analysis indicated that RPS24 exhibited a higher percentage of coverage, and was identified with high confidence by sequencing of two unique tryptic peptides (QmVIDVLHPGK and TTPKVIFVFGFR) from RPS24 using LC-MS/MS analysis, as shown by the corresponding spectra (Fig. 8*C*). The presence of RPS24 immunoreactivity in parental HEK293 cells and in cells co-transfected with cMyc-5-HT_2A_R and HA-mGluR2 or HA-mGluR3 was further confirmed through immunoblot assays (Fig. S6).

**Figure 9:**
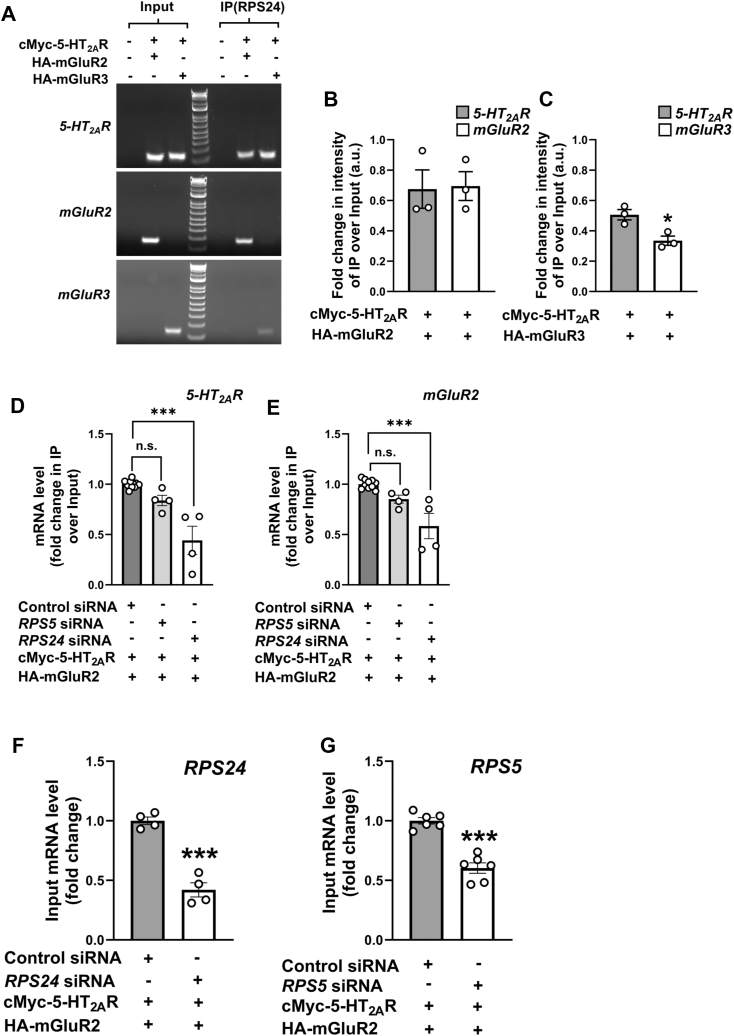
**RPS24 facilitates the interaction between *5-HT*_*2A*_*R* and *mGluR2* transcripts.***A*–*C*, HEK293 cells were co-transfected with pcDNA3.1-cMyc-5-HT_2A_R, and pcDNA3.1-HA-mGluR2 or pcDNA3.1-HA-mGluR3. Representative RT-PCR products for *5-HT*_*2A*_*R*, *mGluR2* and *mGluR3* transcripts from HEK293 cells before (Input) and after immunoprecipitation (IP) using an anti-RPS24 antibody (*A*). Quantification of IP/input band intensities (n = 3 independent experiments) (*B* and *C*). *D* and *E*, HEK293 cells were transfected with non-targeting siRNA, *RPS24* siRNA or *RSP5* siRNA. Forty-eight hours after siRNA transfection, cells were transfected with pcDNA3.1-c-Myc-5-HT_2A_R and pcDNA3.1-HA-mGluR2 constructs. RIP assays were carried out 24 h following DNA transfection using an anti-HA antibody. Subsequently, the RNP complexes underwent processing for RNA isolation and RT-qPCR assays for the detection of *5-HT*_*2A*_*R* (*D*) and *mGluR2* (*E*) transcripts. Data are shown as fold change of IP/input relative to control siRNA (n = 4 independent samples). *F*, HEK293 cells were transfected with non-targeting siRNA or *RPS24* siRNA. Forty-eight hours after siRNA transfection, cells were co-transfected with pcDNA3.1-cMyc-5HT_2A_R and pcDNA3.1-HA-mGluR2 constructs. RNA extractions were carried out 24 h following DNA transfection in Input samples. *RPS24* mRNA was assessed by RT-qPCR (n = 4 independent samples). *G*, HEK293 cells were transfected with non-targeting siRNA or *RPS5* siRNA. Forty-eight hours after siRNA transfection, cells were co-transfected with pcDNA3.1-cMyc-5HT_2A_R and pcDNA3.1-HA-mGluR2 constructs. RNA extractions were carried out 24 h following DNA transfection in Input samples. *RPS5* mRNA was assessed by RT-qPCR (n = 6 independent samples). Unpaired two-tailed Student’s *t* test (*B*, *C*, *F*, *G*), and one-way ANOVA followed by Bonferroni’s *post hoc* test (*D* and *E*) (∗*p* < 0.05, ∗∗∗*p* < 0.001). Data show mean ± s.e.m.

To elucidate the potential involvement of RPS24 in facilitating the interaction between *5-HT*_*2A*_*R* and *mGluR2* transcripts, parental HEK293 cells and cells co-transfected with cMyc-5-HT_2A_R and either HA-GluR2 or HA-mGluR3 were subjected to RIP assays employing an anti-RPS24 antibody. Subsequently, the isolated RNP complexes underwent processing for RNA extraction and RT-PCR assays. Interestingly, the transcripts of *5-HT*_*2A*_*R* and *mGluR2* were more prominently detected in RNP complexes immunoprecipitated by anti-RPS24, as opposed to *mGluR3* transcripts (Fig. 9*A*). Quantitative analysis of the RT-PCR band intensities revealed significant differences in *mGluR3* intensity compared to *mGluR2* or *5-HT*_*2A*_*R* (Fig. 9, *B* and *C*). Additionally, siRNA-mediated knockdown of *RPS24* in HEK293 cells led to a significant reduction in the ability of the anti-HA antibody to pull down HA-mGluR2-associated mRNA transcripts, as determined by RT-qPCR assays (Fig. 9, *D*–*F*). This effect was further validated using a second, independent siRNA targeting a different region of the *RPS24* gene (Fig. S7), and was not observed upon siRNA-mediated knockdown of another ribosomal protein associated with the small subunit *RPS5* (Fig. 9, *D*, *E* and *G*), which further highlights the potentially selective and crucial role of RPS24 in the co-translational assembly of *5-HT*_*2A*_*R* and *mGluR2* transcripts.

### Co-localization of 5-HT_2A_R and mGluR2 in the maturation pathway

**Figure 10:**
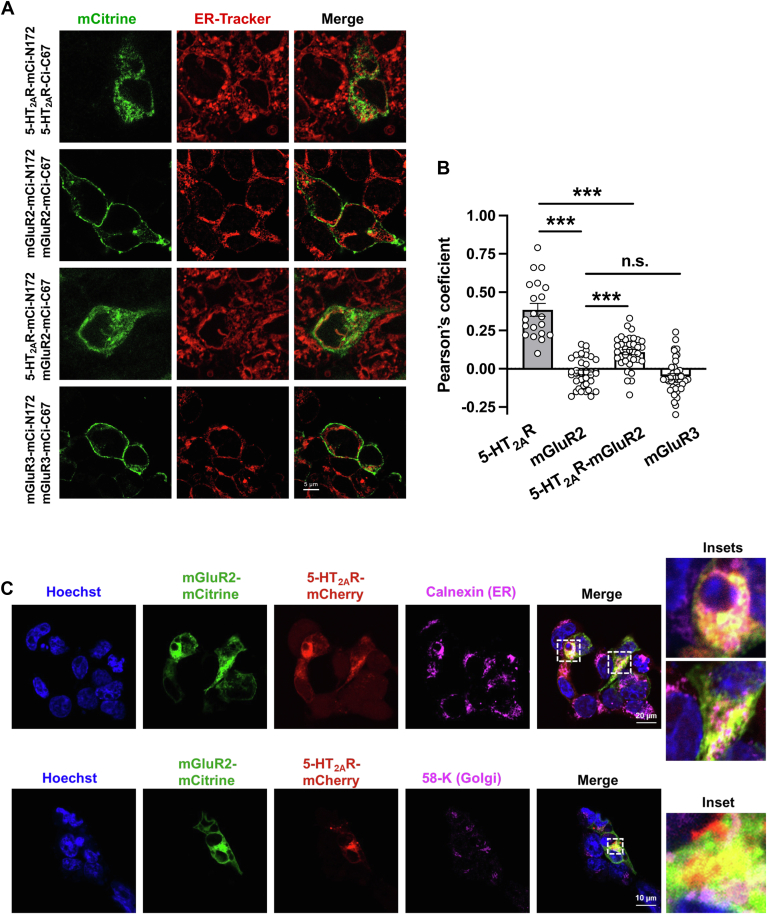
**Co-localization of 5-HT_2A_R and mGluR2 along the maturation pathway.***A* and *B*, BiFC signal in HEK293 cells transfected to co-express 5-HT_2A_R-mCitrine-N172 and 5-HT_2A_R-mCitrine-C67, mGluR2-mCitrine-N172 and mGluR2-mCitrine-C67, 5-HT_2A_R-mCitrine-N172 and mGluR2-mCitrine-C67, or mGluR3-mCitrine-N172 and mGluR3-mCitrine-C67 and stained with ER-Tracker. Representative confocal micrographs (*A*). Pearson’s coefficient colocalization analysis of mCitrine and ER-Tracker signals (n = 20–38 regions of interest in three independent experiments) (*B*). *C*, confocal micrographs of HEK293 cells transiently transfected with pcDNA3.1-cMyc-5-HT_2A_R-mCherry (*red*) and HA-mGluR2-mCitrine (*green*), and stained with anti-calnexin (*upper panel, magenta*) or anti-58-K (*lower panel, magenta*) and secondary antibodies, and imaged to detect mCherry, mCitrine, anti-calnexin, and anti-58-K. Nuclei were stained in *blue* with Hoechst. Enlarged images are shown in the insets of the merged *panels*. Scale bars: 5 μM (*A*), 20 μM (*C*, *top panel*), 10 μM (*C*, *bottom panel*). One-way ANOVA followed by Bonferroni’s *post hoc* test (*B*) (∗∗∗*p* < 0.001; ns, not significant). Data show mean ± s.e.m.

The observation that *5-HT*_*2A*_*R* and *mGluR2* transcripts associate prior to the formation of fully-functional GPCR complexes prompted us to investigate their subcellular localization along the maturation pathway immediately following their co-translational assembly on cytosolic RBP-RNA complexes. To examine whether direct interactions between 5-HT_2A_R and mGluR2 occur during receptor maturation, we employed bimolecular fluorescence complementation (BiFC), a technique that enables visualization of protein–protein interactions in live cells (47). BiFC signal was detected in cells co-transfected with 5-HT_2A_R fused to either the N-terminal (mCi-N172) or C-terminal (mCi-C67) fragment of mCitrine (Fig. 10*A*), confirming the ability of 5-HT_2A_R to form homomeric complexes (29, 48). Consistent with previous studies reporting partial localization of 5-HT_2A_R with endoplasmic reticulum (ER) markers (49, 50), our data showed greater co-localization of BiFC signal with ER-Tracker in cells expressing 5-HT_2A_R-mCi-N172 and 5-HT_2A_R-mCi-C67 than in those expressing mGluR2-mCi-N172 and mGluR2-mCi-C67 (Fig. 10, *A* and *B*). Interestingly, ER co-localization of the BiFC signal was significantly reduced in cells co-expressing 5-HT_2A_R-mCi-N172 and mGluR2-mCi-C67 compared to 5-HT_2A_R homomeric pairs (Fig. 10, *A* and *B*). As a result, the observed ER localization pattern appeared intermediate between that of 5-HT_2A_R–5-HT_2A_R and mGluR2–mGluR2 or mGluR3–mGluR3 BiFC pairs (Fig. 10, *A* and *B*). Similar results were obtained following immunostaining with antibodies against endogenous ER (Calnexin) and Golgi (58K) markers (Fig. 10*C*), further supporting localization of the 5-HT_2A_R–mGluR2 heterocomplex to these subcellular compartments.

### Transcriptional association of 5-HT_2A_R and mGluR2 in mouse frontal cortex

**Figure 11:**
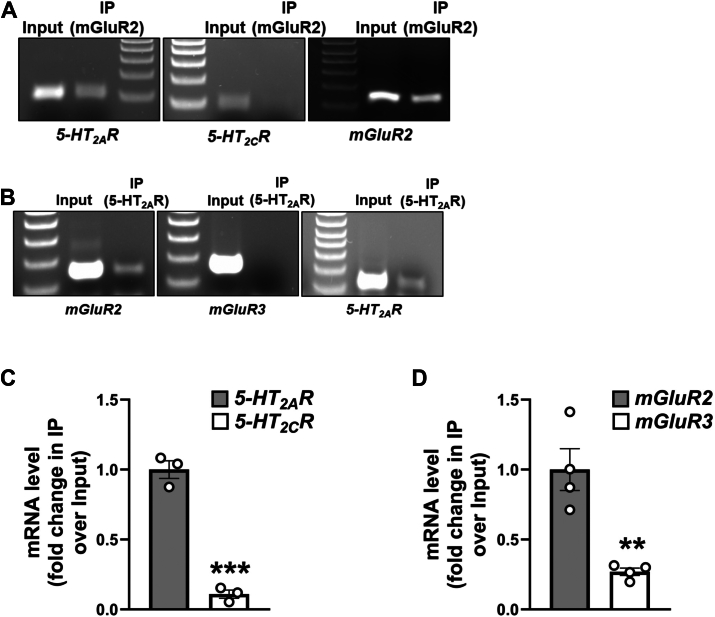
**Co-translational assembly of *5-HT*_*2A*_*R* and *mGluR2* mRNAs in mouse frontal cortex.***A* and *B*, images show representative RT-PCR products for *5-HT*_*2A*_*R*, *5-HT*_*2C*_*R*, *mGluR2* and *mGluR3* transcripts from mouse frontal cortex samples before (Input) and after immunoprecipitation (IP) using anti-mGluR2 (*A*) or anti-5-HT_2A_R (*B*) antibodies. Data are representative from three independent experiments (*A* and *B*). *C* and *D*, mouse frontal cortex samples were subjected to RIP assays employing anti-mGluR2 (*C*) or anti-5-HT_2A_R (*D*) antibodies. Subsequently, the RNP complexes underwent processing for RNA isolation and RT-qPCR assays for the detection of *5-HT*_*2A*_*R*, *5-HT*_*2C*_*R*, *mGluR2,* and *mGluR3* transcripts. Data are shown as fold change of IP/input (n = 3–4 independent samples) (*C* and *D*). Unpaired two-tailed Student’s *t* test (*C* and *D*) (∗∗*p* < 0.01, ∗∗∗*p* < 0.001). Data show mean ± s.e.m.

To investigate the transcript association of *5-HT*_*2A*_*R* and *mGluR2* mRNAs within an endogenous system, we conducted RIP assays using frontal cortex samples from male mice. An anti-mGluR2 antibody directed toward the N-terminus was employed to immunoprecipitate the nascent mGluR2 polypeptide, followed by the assessment of associated transcripts through RT-PCR. Our results indicate that in mouse frontal cortex samples, like in HEK293 cells, nascent mGluR2 polypeptides were in complex with *5-HT*_*2A*_*R* and *mGluR2* transcripts encoding protomers of the 5-HT_2A_R-mGluR2 heterocomplex (Fig. 11*A*). Specificity of this interaction was confirmed by the absence of RT-PCR signal with *5-HT*_*2C*_*R* primers (Fig. 11*A*). A complementary RIP assay using an antibody targeting the N-terminus of 5-HT_2A_R similarly revealed its association with both *5-HT*_*2A*_*R* and *mGluR2* transcripts, but not with *mGluR3* (Fig. 11*B*), further confirming selective transcript co-association within the heteromeric complex. These findings were independently validated by RT-qPCRs in separate animal cohorts (Fig. 11, *C* and *D*).

**Figure 12:**
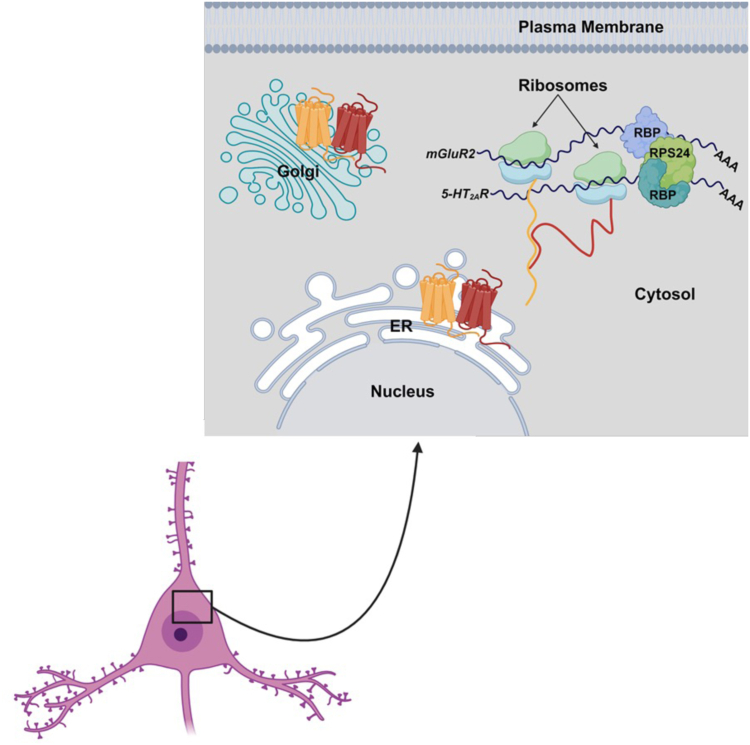
**Schematic representation of a working model for the mechanism of *5-HT*_*2A*_*R* and *mGluR2* transcript association and subcellular localization of the 5-HT_2A_R-mGluR2 heterocomplex during maturation.** Following co-translational association within RNP complexes containing RPS24, 5-HT_2A_R, and mGluR2, they are trafficked to the ER as part of a protein complex in frontal cortex pyramidal neurons. Subsequently, this GPCR heterocomplex is translocated to the Golgi apparatus and directed to the cell membrane or other cellular localizations.

Together, these results suggest that RPS24 promotes the assembly of *5-HT*_*2A*_*R* and *mGluR2* mRNA transcripts in the cytosol. Following this assembly, the fully translated 5-HT_2A_R and mGluR2 components traffic along the maturation pathway as a GPCR heterocomplex. This transcript association occurs independent of their mRNA-to-protein translation. However, it remains unknown whether RPS24 engages directly with these mRNA transcripts, or alternatively if RPS24 functions as an integral component of a multi-protein complex that facilitates the confluence of these transcripts during the process of translation (Fig. 12).

## Discussion

In this study, we have elucidated the pivotal role of 5-HT_2A_R in governing the transcription and translation processes of mGluR2. Suppression of *5-HT*_*2A*_*R* expression using siRNA in HEK293 cells transiently transfected with 5-HT_2A_R and mGluR2 yielded a significant inhibition in the expression of *mGluR2* and its corresponding protein levels, a cross-regulatory event that was not observed in cells co-transfected with 5-HT_2A_R and mGluR3. The observed concurrent decrease in one transcript following the siRNA-induced downregulation of the other implied a potential physical association between the two transcripts. This physical association was further substantiated by RIP assays isolating RNP complexes. Thus, immunoprecipitation of either 5-HT_2A_R or mGluR2, using their tag-specific antibodies, resulted in detection of both transcripts associated with RBPs within RNP complexes containing RPS24 as a pivotal contributor to this molecular assembly. The specificity of this interaction was confirmed by the absence of co-translational association in cells co-transfected with 5-HT_2C_R and mGluR2, or 5-HT_2A_R and mGluR3. To address concerns regarding potential experimental artifacts arising from overexpression, and to evaluate potential co-translational association in a native tissue system, this finding was subsequently validated in mouse frontal cortex tissue samples, where immunoprecipitation of RNP complexes using antibodies against either mGluR2 or 5-HT_2A_R successfully pulled down both *mGluR2* and *5-HT*_*2A*_*R* transcripts, but not *5-HT*_*2C*_*R* or *mGluR3*, supporting the specificity of the interaction.

RNP complexes represent macromolecular entities comprising RBPs and their target RNAs. The reciprocal regulation of RNAs and RBPs occurs within this intricate complex, where RBPs contribute to RNA processes such as localization, stability, processing, modification and translation. Conversely, RNAs can modulate RBP functions, localization, interactions and stability (51, 52). Considering the co-translational association process observed among the *5-HT*_*2A*_*R* and *mGluR2* transcripts, one of our principal objectives was focused on the identification of potential RBPs that may be responsible for this mRNA association. Our mass spectrometry analysis identified two ribosomal RBPs—namely RPS23 and RPS24—in cells co-transfected with *5-HT*_*2A*_*R* and *mGluR2*, or mGluR2 alone; but not in cells mock-transfected or transfected with mGluR3 alone. Based on a higher protein sequence coverage, we focused our efforts on RPS24, and, using an anti-RPS24 antibody, we were able to immunoprecipiate from RNP preparations transcripts encoding both *5-HT*_*2A*_*R* and *mGluR2*, as well as a scarcer fraction of m*GluR3* mRNA, which suggests that the *5-HT*_*2A*_*R* and *mGluR2* mRNA transcripts form part of the same RNP multimer. In shaping the structure of RNA molecules, both intramolecular and intermolecular RNA–RNA interactions (RRIs) assume pivotal roles. These interactions occur either intramolecularly within a single RNA molecule or intermolecularly through engagements among distinct RNA molecules (53), and can be mediated *via* either base pairing or RBP intermediates (54). Additionally, the interactions of RBPs with RNA can range from individual protein-RNA element interactions to the intricate assembly of multiple RBPs and RNA molecules, exemplified by the spliceosome (55). Several neurogenetic disorders, such as Fragile X syndrome, Spinal muscular atrophy, and Paraneoplastic syndromes arise as consequence of mutations in RBPs (56, 57). The RNA binding domains within RBPs serve as the functional component responsible for RNA binding. Among the numerous RNA-binding domains elucidated to date, RNA Recognition Motifs (RRMs) stand out as the most prevalent and extensively investigated RNA-binding domain. While the primary role of the *RPS24* gene is to provide instructions for synthesizing various ribosomal proteins, recent studies also suggest that RPS24 may have alternative cytoplasmic functions, such as participating in chemical signaling pathways, regulating cell division and controlling apoptosis. As one example, siRNA-mediated knockdown of *RPS24* resulted in deficits in the biosynthesis of the 40S subunit of the ribosomes, the subunit that binds RNA and mediates translation (58). Further investigation will be necessary to determine whether RPS24 directly binds the *5-HT*_*2A*_*R* and *mGluR2* mRNA transcripts, or alternatively, if RPS24 is a part of a multi-protein complex that facilitates the association of these transcripts during translation. Moreover, our data do not exclude the possibility that RPS24 influences *mGluR3* transcription through alternative pathways in neuronal and glial cell types.

Our previous data using GPCR protein chimeric constructs showed that three alanine residues located in the intracellular half of the transmembrane four of the mGluR2 were necessary to form a receptor complex with 5-HT_2A_R (26, 28). Current data indicate that mRNA sequences encoding both the VFT domain and the transmembrane portion of the *mGluR2* are sufficient by themselves to associate with the *5-HT*_*2A*_*R* mRNA without requiring their respective translated polypeptides. Although interesting, further investigation will be essential to elucidate the specific positions within these two separate mRNAs that are recognized by RPS24 and/or other RBPs, thereby enabling the inclusion of the two coding sequences responsible for the translation of *5-HT*_*2A*_*R* and *mGluR2* within the same RNA-protein complex, as well as the stoichiometry of these RNP assemblies.

An important finding is the lack of effect of *mGluR2* siRNA on *5-HT*_*2A*_*R* expression, whereas *5-HT*_*2A*_*R* siRNA leads to a reduction in both *mGluR2* transcription and mGluR2 translation in cells co-expressing these two neurotransmitter GPCRs. These *in vitro* findings replicate previous transcriptional and epigenetic observations in mouse frontal cortex samples; as *mGluR2* mRNA expression and mGluR2 density assessed by RT-qPCR and radioligand binding assays with the mGluR2/3 antagonist [^3^H]LY341495, respectively, were downregulated in the frontal cortex *5-HT*_*2A*_*R-KO* mice (43); whereas 5-HT_2A_R expression (mRNA and binding with the 5-HT_2A_R antagonist [^3^H]ketanserin) was unaffected in the same cortical region of *mGluR2-KO* animals (23). While our current findings suggest a translation-independent association of mRNAs encoding *5-HT*_*2A*_*R* and *mGluR2*, further work needs to be completed to clarify the mechanisms by which this unidirectional transcriptional crosstalk occurs in both *in vitro* and *in vivo* models.

We also observed that 5-HT_2A_R and mGluR2 co-localize with markers of intracellular components of the maturation pathway, including the ER and Golgi apparatus. Building on our previous findings revealing that the effect of 5-HT_2A_R on localization of mGluR2 within endosomal compartments required GPCR heteromerization (29), our current data extend this analysis by suggesting physical proximity between 5-HT_2A_R and mGluR2 within intracellular compartments of maturation pathway. For the most part, GPCRs are primarily localized at the cell surface, especially in cells not exposed to an agonist (59). However, certain GPCRs, including the 5-HT_2A_R (29, 50, 60, 61), have a significant intracellular presence. Signal sequences are crucial in the initial stages of intracellular transport of GPCRs. They participate in targeting nascent chains to the ER membrane, initiating the integration of newly synthesized peptides into this compartment (62). Intriguingly, unlike its counterpart the 5-HT_2C_R (63), the N-terminal of the 5-HT_2A_R lacks a putative cleavable signal peptide. Further research will be necessary to better understand the potential role of this mRNA association in the biosynthesis and maturation of the 5-HT_2A_R-mGluR2 heterocomplex (64).

In conclusion, our collective data indicate an association between mRNA transcripts encoding 5-HT_2A_R and mGluR2 in both mammalian cells and mouse frontal cortex, along with their corresponding nascent polypeptides. This association is primarily facilitated by a complex of RBPs, with RPS24 among the key contributors. To the best of our knowledge, this represents the first example of translation-independent association of *GPCR* mRNAs, and these findings may provide a new route to modulate neural transcriptional plasticity processes.

## Experimental procedures

### Plasmid construction

All plasmids described in this study carried transgenes encoding human *GPCR*s. The constructs pcDNA3.1-cMyc-5-HT_2A_R-mCherry, pcDNA3.1-5-HT_2C_R-cMyc-mCitrine, pcDNA3.1-HA-mGluR2-mCitrine, pcDNA3.1-HA-mGluR3-mCitrine, pcDNA3.1-HA-mGluR2-TM, pcDNA3.1-HA-mGluR2-TAG-mCitrine, pcDNA3.1-5-HT_2A_R-mCitrine-N172, pcDNA3.1-5-HT_2A_R-mCitrine-C67, pcDNA3.1-mGluR2-mCitrine-N172 and pcDNA3.1-mGluR2-mCitrine-C67 have previously been described (24, 26, 27, 28). The construct pcDNA3.1-SNAP-mGluR2-HA was kindly donated by Dr Joshua Lewitz at Weill Cornell University. The pcDNA3.1-HA-VFT-mGluR2 construct was prepared by PCR amplification from the full length pcDNA3.1-HA-mGluR2-mCitrine construct as template and the primers 5′-CGCGGCTAGCATGGTCCTTCTGTTGATCCTGTC-3′ and 5′-CGCGGGATCCTTACAGTTCGAAGCAGCCAGTCAG-3′. The pcDNA3.1-TAA-HA-VFT-mGluR2 construct was prepared by PCR amplification from the full length pcDNA3.1-TAA-HA-mGluR2-mCitrine construct as template and the primers 5′- CGCGGCTAGCTAAGTCCTTCTGTTGATCCTGTC-3′ and 5′-CGCGGGATCCTTACAGTTCGAAGCAGCCAGTCAG-3′. All PCRs were performed using Q5 High-Fidelity DNA Polymerase (NEB) in a Mastercycler Ep Gradient Auto thermal cycler (Eppendorf). Cycling conditions were 25 cycles of 98 °C for 10 s, 55 °C for 30 s, and 72 °C for 20 s/kb of amplicon, with an initial denaturation/activation of 98 °C for 30 s and a final extension of 72 °C for 2 min. The final PCR products were digested using NheI and BamHI restriction enzymes and sub-cloned into the same restriction sites of pcDNA3.1 vector. The pcDNA3.1-mGluR3-mCitrine-N172 and pcDNA3.1-mGluR3-mCitrine-C67 constructs were generated by digesting the mGluR3 construct C-terminally tagged with mCitrine using *NheI* and *NotI*, and subcloning the resulting fragment into the same restriction sites of pcDNA3.1-mCitrine-N172 and pcDNA3.1-mCitrine-C67. Introduction of mutation (ATG to TAA) into the pcDNA3.1-HA-mGluR2-TM and pcDNA3.1-HA-mGluR2-TAG-mCitrine constructs was performed with the QuikChange II Site-Directed Mutagenesis Kit (Agilent, Catalog no. 200523) (for primer pairs, see Table S2). All the constructs were confirmed by DNA sequencing.

### Mammalian cell culture

Human embryonic kidney (HEK293) cells (ATCC: CRL-1573) were maintained in Dulbecco’s modified Eagle’s medium (DMEM) supplemented with 10% dialyzed fetal bovine serum (dFBS) and 1% penicillin/streptomycin (Gibco) at 70 to 80% confluency in a 5% CO_2_ humidified atmosphere.

### Mouse brain samples

On the day of the experiment, male mice (C57BL/6J) were killed by cervical dislocation, and bilateral frontal cortex samples (bregma 1.90–1.40 mm) stored at −80 °C until tissue processing. *5-HT*_*2A*_*R* knockout (*Htr2a*^*−/−*^) mice of C57BL/6J background have been previously described (65). All procedures were conducted in accordance with the National Institutes of Health (NIH) guidelines and were approved by the Virginia Commonwealth University Animal Care and Use Committee.

### RNA interference

For small interfering RNA (siRNA) assays, cells were transfected with non-targeting siRNA (Santa Cruz Biotechnology, Inc), *5-HT*_*2A*_*R* siRNA, *mGluR2* siRNA, *RPS24* siRNA, or *RPS5* siRNA (for siRNA target sequences, see Table S3) (Sigma) using Oligofectamine (Invitrogen), following manufacturer’s instructions. Forty-eight hours after siRNA transfection, cells were transfected with pcDNA3.1-cMyc-5-HT_2A_R-mCherry, pcDNA3.1-HA-mGluR2-mCitrine, and/or pcDNA3.1-HA-mGluR3-mCitrine using Lipofectamine 3000 (Invitrogen). Assays including RNA and protein extraction, and isolation of RNP complexes was carried out 24 h following DNA transfection.

### Ribonucleoprotein complex isolation

RNP complexes were isolated using a RiboCluster Profiler TM RIP-Assay Kit (Medical & Biological Laboratories, catalog no. RN1001), according to manufacturer’s protocol. Briefly, HEK293 cells were transiently co-transfected (1:1 ratio) with pcDNA3.1-cMyc-5-HT_2A_R-mCherry, pcDNA3.1-5-HT_2C_R-cMyc-mCitrine, pcDNA3.1-HA-mGluR2-mCitrine, pcDNA3.1-HA-mGluR3-mCitrine, pcDNA3.1-TAA-HA-mGluR2-TAG-mCitrine, pcDNA3.1-TAA-HA-VFT-mGluR2, pcDNA3.1-TAA-HA-mGluR2-TM and/or pcDNA3.1-SNAP-mGluR2-HA using Lipofectamine 3000. Twenty-four hours post DNA transfection cells were processed for isolation of RNP complexes. For this, immunoprecipitation assays were performed for 3-h at 4 °C using anti-cMyc (Cell Signaling technology, catalog no. 2278), anti-HA (Cell Signaling technology, catalog no. 3724), anti-SNAP (NEB, catalog no. P9310S), anti-RPS24 (Abcam, Catalog no. ab196652), or anti-IgG (RIP-Assay Kit, see above) antibodies. This was followed by RNA isolation, DNase one treatment, cDNA preparation and analysis of target RNAs using RT-PCR and RT-qPCR assays. For polysome dissociation assays, 25 mM EDTA (ethylenediaminetetraacetic acid; Sigma) or vehicle was added to the RIP lysis buffer during the isolation of RNP complexes.

Mouse frontal cortex samples were homogenized using Qiagen TissueRuptor II (120 V, 60 Hz, and Catalog no. 9002755) in the lysis buffer and proceeded as recommended by manufacturer’s protocol (see above). Immunoprecipitation of mGluR2 or 5-HT_2A_R was performed overnight at 4 °C using a mouse monoclonal anti-mGluR2 N-terminal antibody (Abcam, Catalog no. ab15672) or a rabbit polyclonal anti-5-HT_2A_R N-terminal antibody (Immunostar, Catalog no. 24288), followed by RNA isolation, DNase one treatment, cDNA preparation and analysis of target RNAs using RT-PCR and RT-qPCR assays (for primer pair sequences, see Table S2). Specificity of the anti-mGluR2 antibody has been previously reported using *mGluR2-KO* mice (27). For confirmation of specificity of the primary antibody against 5-HT_2A_R in experiments with *5-HT*_*2A*_*R-KO* mice, see Figure S8.

### RNA isolation, RT-PCR, and RT-qPCR

RT-PCR and RT-qPCR assays were performed as previously reported (23, 44), with minor modifications (for primer pair sequences, see Table S2). Briefly, total RNA was isolated using RNeasy Mini Kit (Qiagen, catalog no.74104), according to manufacturer’s protocol. Total RNA (2 μg) was then reverse transcribed using High-Capacity cDNA Reverse Transcription Kit (Applied Biosystems, catalog no. 4374966), according to manufacturer’s instructions.

For RT-PCR assays, cDNA (1:30 dilution) was utilized for 30-cycle three-step PCR in Mastercycler Ep Gradient Auto thermal cycler (Eppendorf) using Q5 High-Fidelity 2× Master Mix (NEB) and 200 nM of each primer. After electrophoresis, DNA bands were visualized under UV light after staining the agarose gel with ethidium bromide. Quantification of DNA band signals was performed using ImageLab (Biorad). To determine the fold-change for a given transcript between two experimental conditions (e.*g*., cMyc-5-HT_2A_R and HA-mGluR2 or HA-mGluR3) in (Fig. 9, *B* and *C*) DNA band intensities were normalized to the intensities of the same transcript in the input band.

For RT-qPCR assays, cDNA (1:400, 1:100, and 1:30 dilutions for cell, mouse Input, and mouse IP samples, respectively) was utilized for 40-cycle three-step PCR using PowerUp SYBR Green Master Mix (Applied Biosystems, catalog number A25742) in an Applied Biosystems QuantStudio 6 Flex Real-Time PCR machine. Each transcript in each sample was assayed two times, and the median threshold cycle (C_T_) was used to calculate the fold change values; using the 2^−cCT^ as we have previously reported (66). The housekeeping gene *GAPDH* was used as an internal control for normalization. In RT-qPCR experiments using total cDNA samples from HEK293 cells (*e.g*., Fig. 1, *A* and *B*), the corrected C_T_ (cC_T_) for a given transcript (*e.g*., *mGluR2*) was calculated as cC_T(*mGluR2*)_ = C_T(*mGluR2*)_ − C_T(*GAPDH*)_. To determine the fold-change for a given transcript, the mean of 2^−cCT^ for that transcript in all the control samples (*e.g*., control siRNA) was determined first (control mean). The fold change for each transcript was then calculated from the cC_T_ as 2^−cCT^/control mean. In RT-qPCR experiments using RIP samples in HEK293 cells (*e.g*., Fig. 3, *E*–*H*) and mouse frontal cortex samples (*e.g*., Fig. 11, *C* and *D*), the corrected C_T_ (cC_T_) for a given transcript (*e.g.,* immunoprecipitated *mGluR2* and input *mGluR2*) was calculated as cC_T(IP_ _*mGluR2*)_ = C_T(IP_ _*mGluR2*)_ − C_T(IP_ _*GAPDH*)_ and cC_T(Input_ _*mGluR2*)_ = C_T(Input_ _*mGluR2*)_ − C_T(Input_ _*GAPDH*)._ After this, 2^−cCT^ (immunoprecipitated) values were normalized to 2^−cCT^ (input) values of the same transcript. To determine the fold-change, the mean of IP/input in all the control samples (*e.g*., control siRNA) was determined first (control mean). The fold change was then calculated for all the experimental conditions as (IP/input)/control mean.

### Immunoblot

Whole cell lysates (Fig. 1, *D* and *F*, Fig. 2, *C*, *F* and *I*, Fig. 3, *I* and *J*, Fig. 5*C*, Fig. 7, *B* and *C*, and Fig. S5) were prepared by disrupting cells using 1× lysis buffer followed by sonication. For RIP preparations (Figs. S2, S3, and S6), cells were harvested and lysed using RIP lysis buffer (see above) for 10 min on ice, followed by centrifugation at 12,000×*g* for 5 min at 4 °C. The resulting supernatant was mixed with 1× Laemmli sample buffer and boiled at 95 °C for 5 min. For nuclear fraction preparations (Fig. S2), HEK293 cells were harvested in cold 1× DPBS and homogenized in Tris-HCl (50 mM, pH 7.4). Western blot experiments in mouse frontal cortex tissue samples were performed as previously reported (44). The homogenate was centrifuged at 1000×*g* for 5 min at 4 °C. The resulting supernatant was discarded and the nuclear pellet was re-suspended in 1× Laemmli sample buffer, sonicated and boiled at 95 °C for 5 min. Equal amounts of protein were separated on an 8% SDS-polyacrylamide gel and transferred to a nitrocellulose blotting membrane. After this, membranes were blocked with 0.1%TBS-T containing 2.5% NFDM and 0.5% BSA, and probed overnight at 4 °C with primary antibodies: anti-cMyc (Cell Signaling technology, catalog no. 2276, 1:1000), anti-HA (Cell Signaling technology, catalog no. 3724, 1:1000), anti-GAPDH (Cell Signaling technology, catalog no. 2118, 1:1000 for cell preparations, and 1:3000 for mouse frontal cortex preparations), anti-Lamin A/C (Cell Signaling technology, catalog no. 4777, 1:2000), anti-α-tubulin (Cell Signaling technology, catalog no. 2125, 1:2000) anti-RPS24 (Abcam, catalog no. ab196652, 1:1000), anti-mGluR2 (catalog no. 76012S, 1:1000), and anti-5-HT_2A_R (Immunostar, catalog no. 24288; 1:1000). Blots were then probed with secondary antibodies (Rabbit IgG HRP Linked Whole Ab, Cytiva NA934-1ML and Mouse IgG HRP Linked Whole Ab, Cytiva NA931-1ML), and immunoreactivity detected with the enhanced chemiluminescence system according to the manufacturer’s instructions. These data compared well with the expected molecular weights of cMyc-5-HT_2A_R-mCherry monomer (∼81 kDa), dimer (∼162 kDa), and oligomers (>250 kDa), 5-HT_2C_R-cMyc-mCitrine monomer (∼79 kDa), dimer (∼158 kDa), and oligomers (>250 kDa), HA-mGluR2-mCitrine monomer (∼123 kDa) and dimer (∼246 kDa), HA-mGluR3-mCitrine monomer (∼126 kDa) and dimer (∼252 kDa), HA-mGluR2-VFT monomer (∼63 kDa), HA-mGluR2-TM monomer (∼60 kDa), GAPDH (∼36 kDa), anti-Lamin A/C (∼65 kDa and ∼70 kDa), α-tubulin (∼50 kDa), RPS24 (∼15 kDa), and mouse 5-HT_2A_R (∼75 kDa). Fold-change in immunoreactivity was calculated from experimental target and loading control in each lane using ImageLab software (Biorad). Data represent the average fold-change values from three independent experiments.

### LC-MS/MS analysis

HEK293 cells were transiently transfected with pcDNA3.1-cMyc-5-HT_2A_R-mCherry, pcDNA3.1-HA-mGluR2-mCitrine and/or pcDNA3.1-HA-mGluR3-mCitrine, or untransfected (mock). Forty-eight hours after post-transfection, cells were processed for RNP complexes isolation and immunoprecipitation using the anti-HA antibody (see above) and then digested using commercially available Preomics iST sample clean-up protocol. Samples containing approximately 1μg-50μg of protein were mixed with 50 μl of lysis buffer, followed by an incubation for 10 min at 95 °C. Samples were then spun at 1000 rpm to eliminate any foam created during sonication. After this, 50 μl of DIGEST solution was added to the mixture, incubated at 37 °C for 3 h and centrifuged at 500 rpm. After digestion, 100 μl of STOP solution was added and mixed properly. The digest was then centrifuged at 3800 rpm for 3 min to ensure complete flow through and washed with 200 μl of WASH 1 and 200 μl of WASH two solution followed by centrifugation after each wash. The cartridge was then placed in a fresh collection tube, and 100 μl of ELUTE solution was added and centrifuged at 3800 rpm for 3 min to ensure complete flow through. This step was repeated once more to ensure maximum recovery. The elutes were then placed in a vacuum evaporator at 45 °C until they dried completely.

LC-MS/MS analysis was performed using a Q-Exactive HF-X (Thermo) tandem mass spectrometer coupled to an Easy nLC 1200 (Thermo) nanoflow UPLC system. The LC-MS/MS system was fitted with an easy spray ion source and an Acclaim PepMap (75 μm × 2 cm) nanoviper C18 (3 μm × 100 Å) pre-column in series with an Acclaim PepMap RSLC (75 μm × 50 cm) C18 2 μm bead size (Thermo). The mobile phase consists of Buffer A (0.1% formic acid in water) and Buffer B (80% acetonitrile in water, 0.1% formic acid). The peptides were injected onto the above column assembly and eluted with acetonitrile/0.1% formic acid gradient at a flow rate of 300 nl/min for over 1.6 h. The nano-spray ion source was operated at 1.9 kV. The digests were analyzed using a data dependent acquisition (DDA) method acquiring a full scan mass spectrum (MS) followed by 15 tandem mass spectra (MS/MS) in the high energy C- trap Dissociation HCD spectra. This mode of analysis produces approximately 50,000 MS/MS spectra of ions ranging in abundance over several orders of magnitude. Not all MS/MS spectra are derived from peptides.

LC-MS/MS datasets data were analyzed in Proteome Discoverer (ver 3.0) using the SEQUEST HT search algorithm and a Custom human and contaminant protein database. Proteins were identified at an FDR <0.01. The m/z (mass-to-charge) ratios were analyzed to identify peptide fragments. Based on this, b ions denote peptide fragments containing the N-terminal of the peptide, whereas y ions correspond to peptide fragments that include the C-terminal. The m/z ratios are presented as the mass of the ion divided by its charge.

### Bimolecular fluorescence complementation

Bimolecular fluorescence complementation (BiFC) and colocalization assays were performed as previously reported (29), with minor modifications. Briefly, approximately 30-h post-transfection, HEK293 cells on coverslips were incubated with ER-Tracker Red (ThermoFisher) following manufacturer’s instructions, then fixed with 3% PFA for 2 min. For colocalization assays, raw 16 bit image files were imported into Fiji (version 2.0.0) with the Coloc two plugin. To assess colocalization, Pearson’s correlation coefficients were calculated based on pixel-intensity correlation in experiments where the experimenter was blinded to the experimental conditions.

### Immunocytochemistry

Approximately 30-h post-transfection, HEK293 cells were fixed with either 4% PFA or ice-cold methanol. Coverslips containing cells were washed thrice with 1x PBS to remove PFA/methanol. Cells were then permeabilized using 0.1% Triton X-100 for 5 min at room temperature and subsequently treated with 5% BSA for 1 h. Primary antibodies targeting endoplasmic reticulum (Calnexin, Invitrogen, Catalog no. MA3-027, 1:500) or Golgi (58K-9, Abcam, Catalog no. ab27043, 1:100) were incubated overnight at 4 °C. After washing thrice with 1x PBS, cells were incubated with goat anti-mouse IgG (H+L) Cross-Adsorbed Secondary Antibody, Alexa Fluor 647 (Invitrogen, Catalog no. A-21235, 1:500) for 1-h at room temperature. Cells were washed thrice with 1x PBS and nuclei were stained with Hoechst 33,342 Solution (Thermo Scientific, Catalog no. 62249, 1:1000 of 1 mg/ml solution) for 10 min at room temperature. After washing twice with 1 × PBS, coverslips were mounted on glass slides using ProLong Diamond Antifade Mountant (Invitrogen, Catalog no. P36961).

Images of fixed cells were acquired using a Carl Zeiss Axio Observer LSM 710 laser scanning confocal microscope with a Plan-Apochromat 63 × /1.40 Oil DIC M27 objective. Hoechst 33342 was excited by a 405-nm blue diode, mCitrine by 488-nm multiline argon laser, mCherry by 561-nm green diode laser, and AF-647 was excited with a 633-nm HeNe laser line. Emission signals were acquired in the same order using the following main beam splitter/dichroic beam splitter (MBS/DBS) and emission filters (EF) sets: InVis 405/Mirror EF, 410 to 483 nm; 488/Mirror EF, 495 to 554 nm; 458/561/633/Mirror EF, 582 to 632 nm and 635 to 730 nm for AF-647. Pinhole was kept constant at one airy unit (AU). After acquisition, images were processed using Fiji software.

### Statistical analysis

Statistical significance was assessed by unpaired Student’s *t* test, and one-way or two-way ANOVA followed by Bonferroni’s *post hoc* test. Datapoints were excluded based on previously established criterion and were set to ± 2 SD from the group mean. All statistical analyses were performed with GraphPad Prism software, version 10 (Graph-Pad Software, Inc), and comparisons were considered statistically significant if *p* < 0.05. Data are presented as mean ± s.e.m.

## Data availability

The data that support the findings in this study are available from the corresponding author upon reasonable request.

## Supporting information

This article contains supporting information.

## Conflict of interest

The authors declare the following financial interests/personal relationships which may be considered as potential competing interests. J. G.-M. has previously received research support from Terran Biosciences. S. S. declares no conflict of interest.

## Supporting information

Saha_supporting_figure_legends

Saha_supporting_figures

Saha_supporting_tableS1

Saha_supporting_tableS2

Saha_supporting_tableS3
