# Supplementary material for: Translation-independent association of mRNAs that encode protomers of the 5-HT2A-mGlu2 receptor complex
Source: J Biol Chem. 2025 Jun 26;301(8):110427. doi: 10.1016/j.jbc.2025.110427 (PMC12305237; doi:10.1016/j.jbc.2025.110427)
Supplement: Saha_supporting_figure_legends [file mmc1.docx]

**Supporting Figure legends**

**Figure S1. Validation assays using siRNAs targeting a different region of the *5-HT_2A_R* gene.** (*A-C*) HEK293 cells were transfected with non-targeting siRNA or *5-HT_2A_R* siRNA. Forty-eight hours after siRNA transfection, cells were transfected with pcDNA3.1-cMyc-5-HT_2A_R (*A*), or co-transfected with pcDNA3.1-cMyc-5-HT_2A_R and pcDNA3.1-HA-mGluR2 (*B*) or pcDNA3.1-HA-mGluR3 (*C*). RNA extractions were carried out 24 h following DNA transfection. 5-HT_2A_R (*A*), *mGluR2* (*B*), and *mGluR3* (*C*) mRNA was assessed by RT-qPCR (n = 4 independent samples). Unpaired two-tailed Student’s *t*-test (*A-C*) (**p < 0.01; ***p < 0.001). Data show mean ± s.e.m.

**Figure S2. Validation of cytosolic fractionation of RNP complexes using RIP Assay**. (*A, B*) Immunoblot with an anti-α-Tubulin antibody (cytosolic marker) (*A*) and an anti-Lamin A/C antibody (nuclear marker) (*B*) in RIP cell lysate and nuclear preparations of HEK293 cells.

**Figure S3. HA and cMyc immunoreactivity in both Input and RIP samples.** (*A*) HEK293 cells were co-transfected with pcDNA3.1-HA-mGluR2, and pcDNA3.1-cMyc-5-HT_2A_R or pcDNA3.1-5-HT_2C_R-cMyc constructs, or mock. Images show representative immunoblots from RIP samples before (Input) and after immunoprecipitation (IP) using an anti-HA antibody. (*B*) HEK293 cells were co-transfected with pcDNA3.1-cMyc-5-HT_2A_R, and pcDNA3.1-HA-mGluR2 or pcDNA3.1-HA-mGluR3 constructs, or mock. Images show representative immunoblots from RIP samples before (Input) and after immunoprecipitation (IP) using an anti-c-Myc antibody.

**Figure S4. Assays with non-immune immunoglobulin (IgG) to control for RIP selectivity.** (*A-B*) HEK293 cells were co-transfected with pcDNA3.1-cMyc-5-HT_2A_R and pcDNA3.1-HA-mGluR2 constructs. RIP assays were carried out 24 h following DNA transfection using IgG antibody. Subsequently, input and IP samples underwent processing for RNA isolation and RT-qPCR assays for the detection of *5-HT_2A_R* (*A*) and *mGluR2* (B) transcripts. Data are shown as fold change over input (n = 6 independent samples). (*C-D*) HEK293 cells were co-transfected with pcDNA3.1-5-HT_2C_R-cMyc and pcDNA3.1-HA-mGluR2 constructs. RIP assays were carried out 24 h following DNA transfection using IgG antibody. Subsequently, input and IP samples underwent processing for RNA isolation and RT-qPCR assays for the detection of *5-HT_2C_R* (*C*) and *mGluR2* (*D*) transcripts. Data are shown as fold change over input (n = 6 independent samples). Unpaired two-tailed Student’s *t*-test (***p < 0.001) (*A-D*). Data show mean ± s.e.m.

**Figure S5. Characterization of TAA-HA-mGluR2-TAG construct.**

Immunoblot with an anti-mGluR2 antibody that targets extracellular loops of mGluR2 in HEK293 cells transiently transfected with pcDNA3.1-HA-mGluR2 or pcDNA3.1-TAA-HA-mGluR2-TAG. Because the immunoblots shown in Fig. 5C and Fig. S5 were performed simultaneously, the same blot probed for the housekeeping protein GAPDH is reused in both panels.

**Figure S6. Endogenous expression of RPS24 in HEK293 cells.** Immunoblot depicting RPS24 immunoreactivity in RIP cell lysates from parental HEK293 cells, as well as cells co-transfected with pcDNA3.1-cMyc-5HT_2A_R, and pcDNA3.1-HA-mGluR2 or pcDNA3.1-HA-mGluR3.

**Figure S7. Validation assays using siRNAs targeting a different region of the *RPS24* gene.** (*A-C*) HEK293 cells were transfected with non-targeting siRNA or *RSP24* siRNA. Forty-eight hours after siRNA transfection, cells were co-transfected with pcDNA3.1-cMyc-5-HT_2A_R and pcDNA3.1-HA-mGluR2. RNA extractions were carried out 24 h following DNA transfection. *RPS24* (*A*), *5-HT_2A_R* (*B*), and *mGluR2* (*C*) mRNA was assessed by RT-qPCR (n = 4 independent samples). Data are shown as fold change in input (*A*) and IP/input relative to control siRNA (*B*, *C*). Unpaired two-tailed Student’s *t*-test (*p < 0.05; **p < 0.01) (*A-C*). Data show mean ± s.e.m.

**Figure S8. Selectivity of the anti-5-HT_2A_R antibody in mouse frontal cortex samples.** Representative immunoblots with anti-5-HT_2A_R and anti-GAPDH antibodies in frontal cortex of wild-type (WT) and *5-HT_2A_R-KO* (KO) mice.
