# Supplementary figures and images for: Translation-independent association of mRNAs that encode protomers of the 5-HT2A-mGlu2 receptor complex
Source: J Biol Chem. 2025 Jun 26;301(8):110427. doi: 10.1016/j.jbc.2025.110427 (PMC12305237; doi:10.1016/j.jbc.2025.110427)

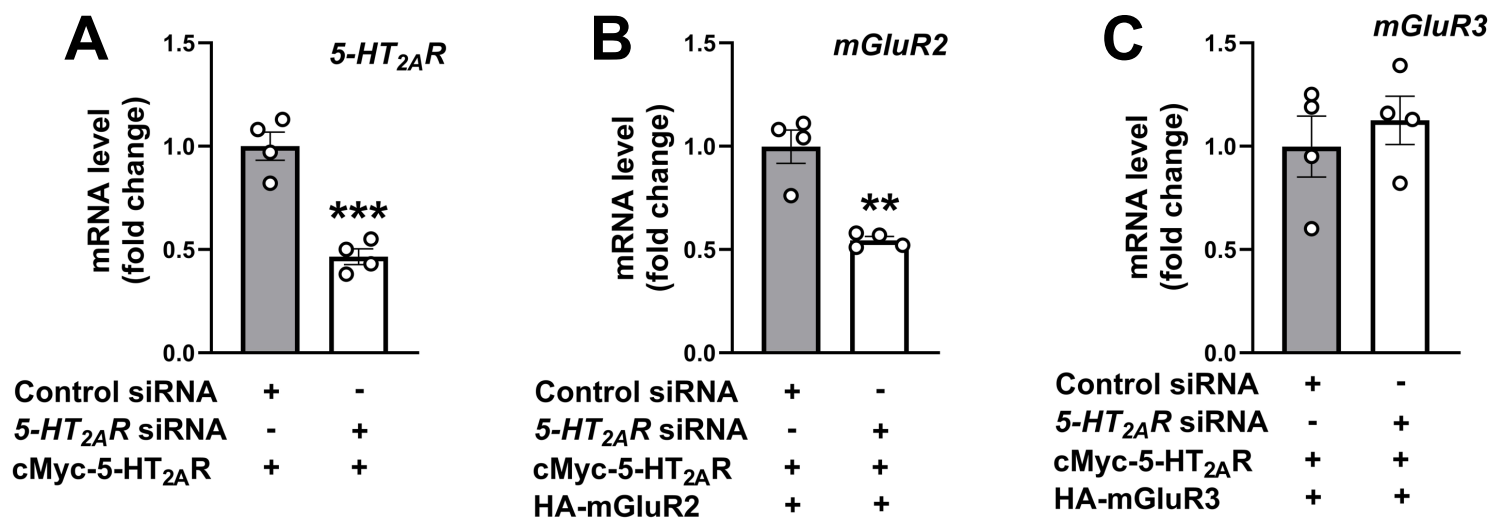

**Figure S1**

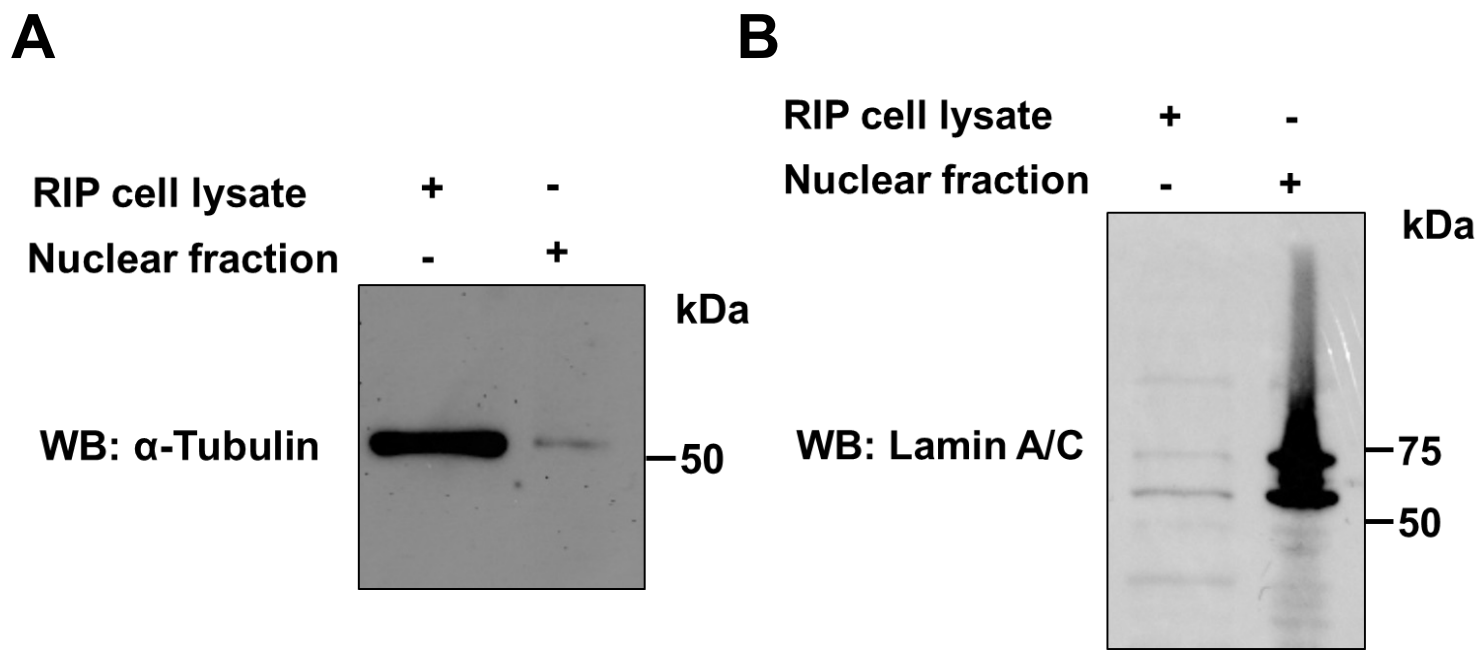

Figure S2

**A**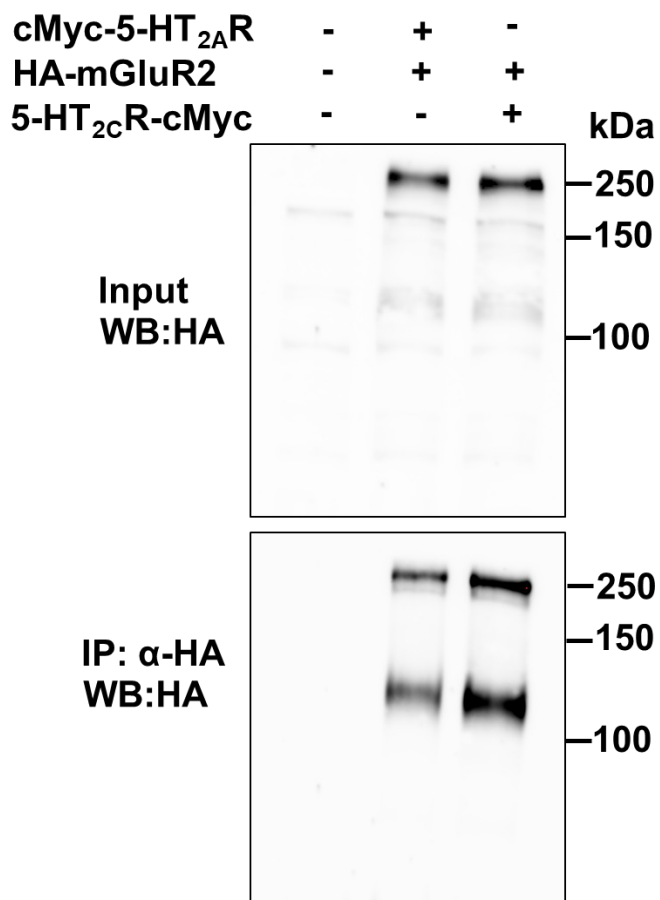**B**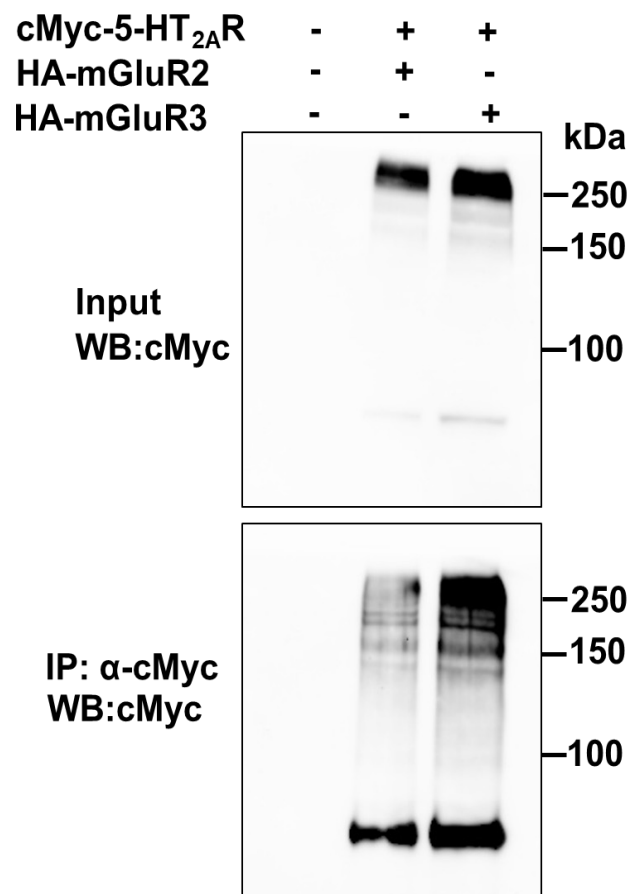**Figure S3**

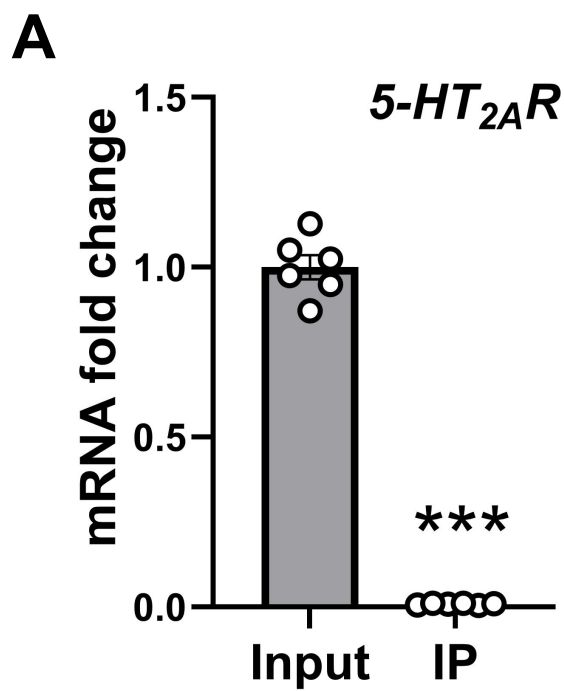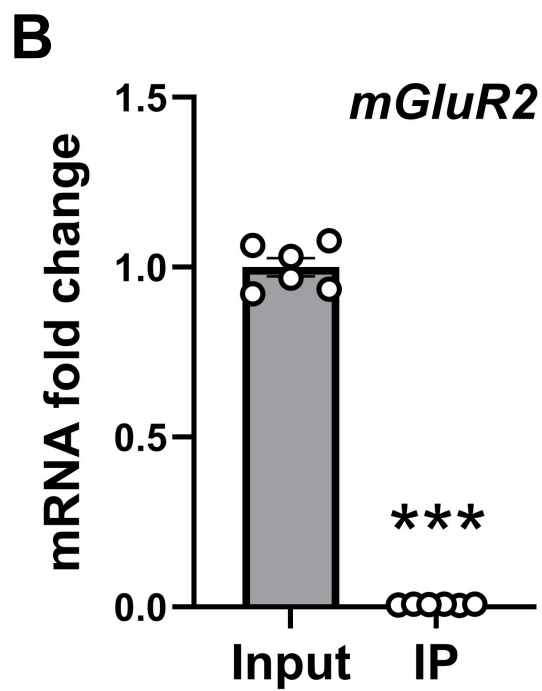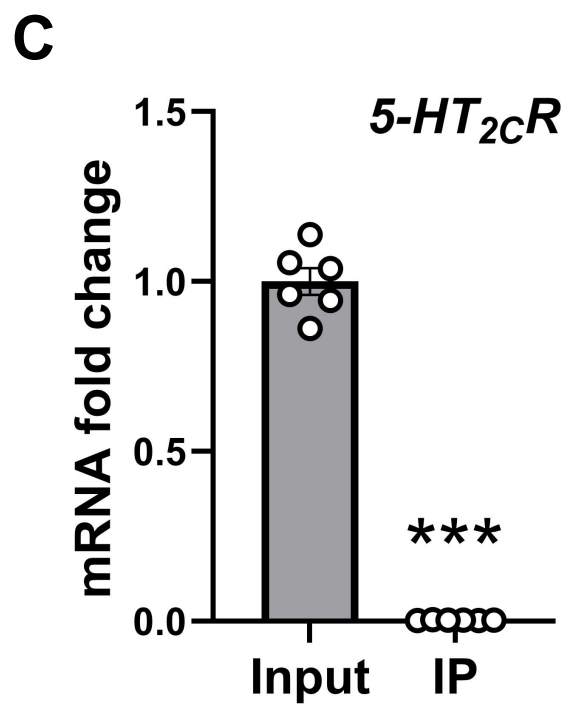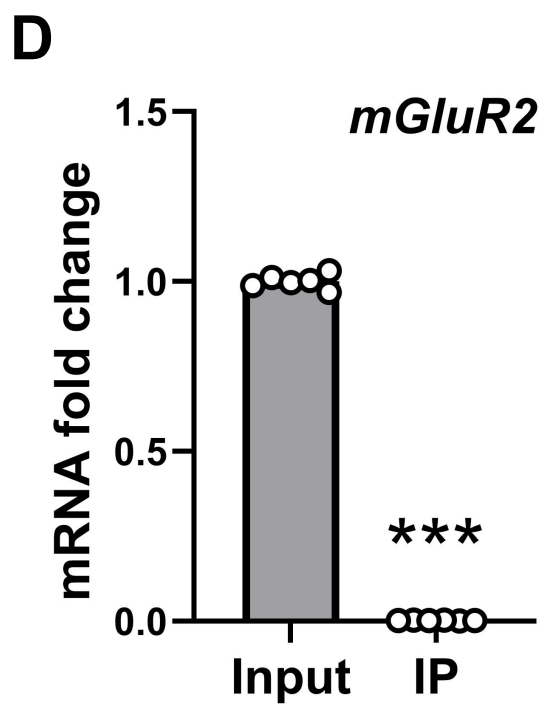

Figure S4

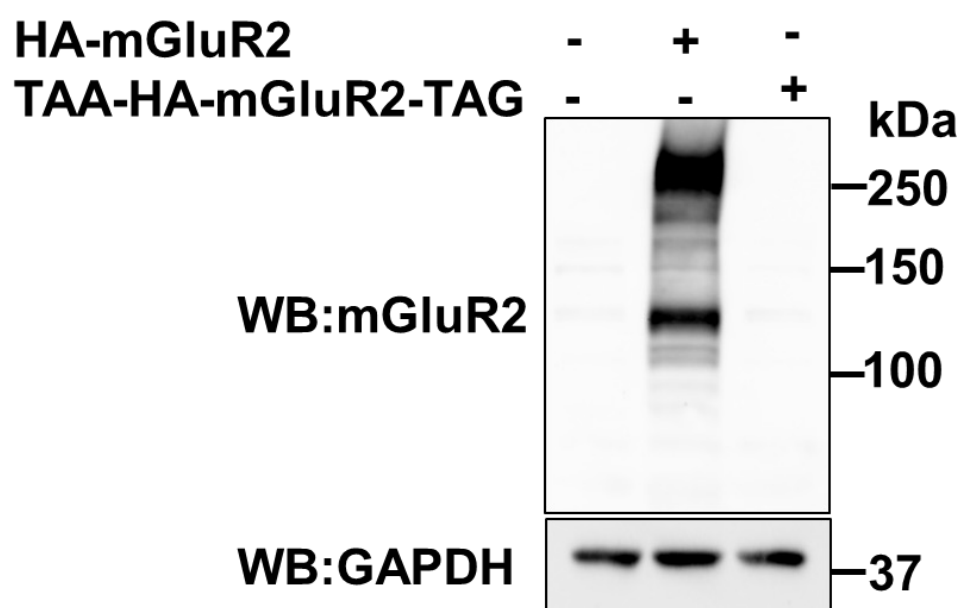

**Figure S5**

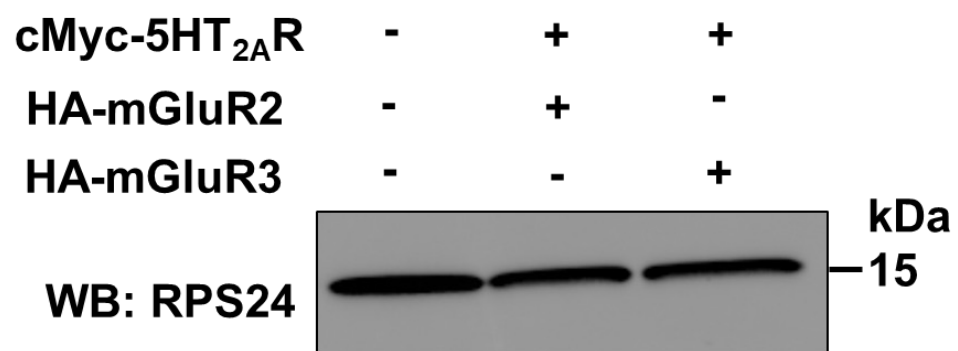

**Figure S6**

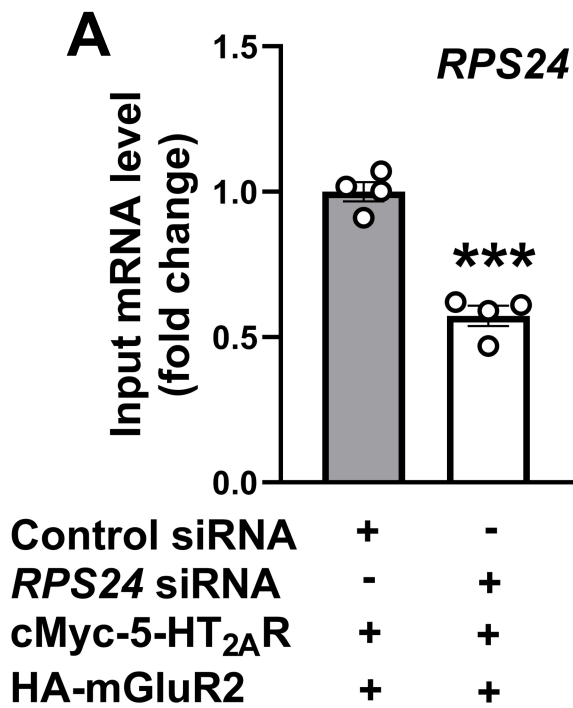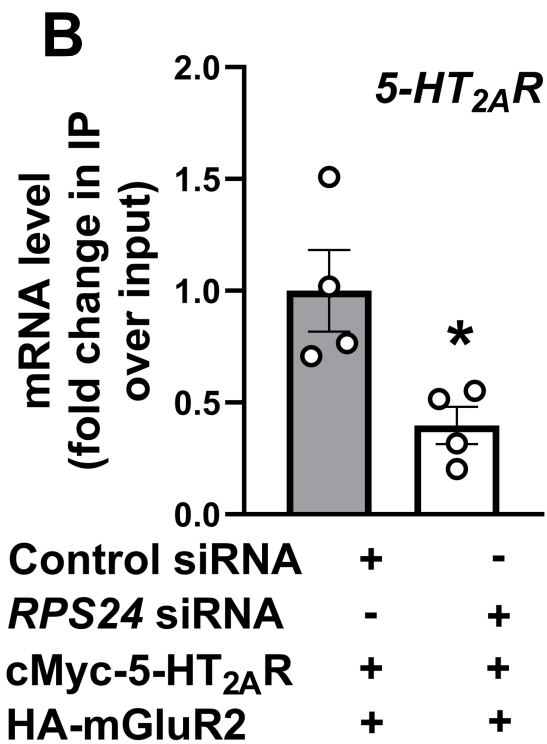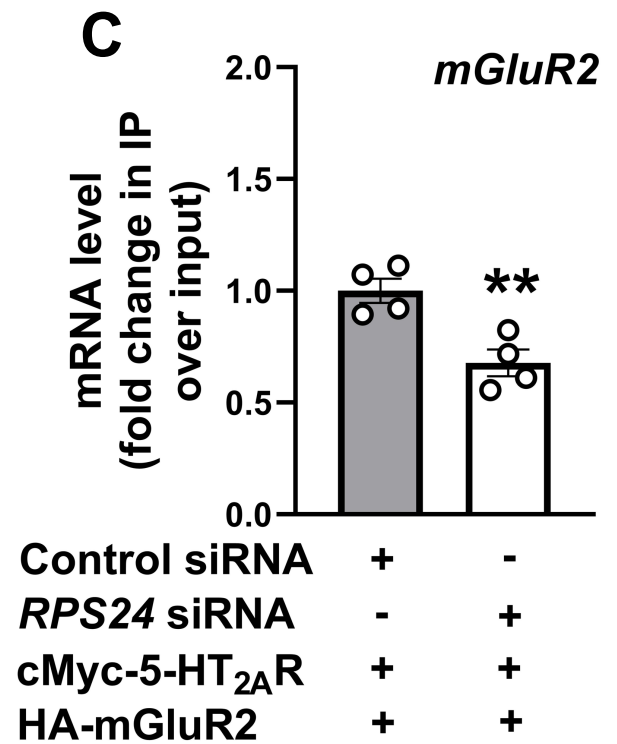

Figure S7

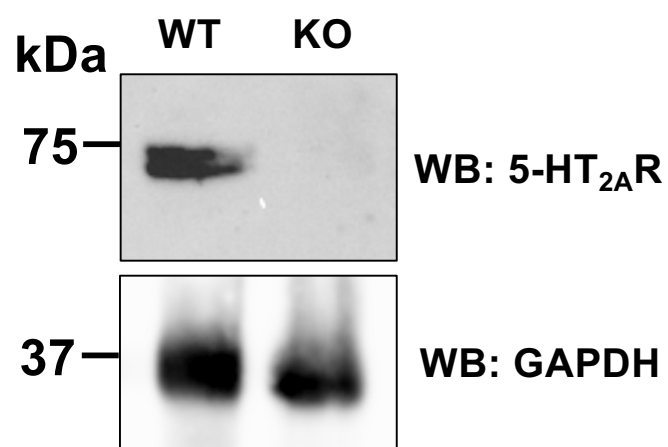

**Figure S8**

Supplement: Saha_supporting_figures [file mmc2.pdf]
