# Supplementary material for: Translation-independent association of mRNAs that encode protomers of the 5-HT2A-mGlu2 receptor complex
Source: J Biol Chem. 2025 Jun 26;301(8):110427. doi: 10.1016/j.jbc.2025.110427 (PMC12305237; doi:10.1016/j.jbc.2025.110427)
Supplement: Saha_supporting_tableS2 [file mmc4.pdf]

Supplementary Table S2:
Primer sequences for RT-PCR, RT-qPCR and site-directed mutagenesis

Gene Name	GenBank	Primer pairs (5'-3')
GRM2 (*mGluR2*) - human	NM_000839.5	**F** CGGTTCCTACAGTGATGTCTCC (VFT)
		**R** TGGCTTGGAAGAAGTCAGGAGG (VFT)
		**F** CCGCATTGCACGCATCTTC (TM)
		**R** GGCCCGAGATAAGTGCCAG (TM)
HTR2A (*5-HT$_{2A}$R*) - human	NM_000621.5	**F** ATCCACCACAGCCGCTTCAACT
		**R** TCATCGGCGAGTAAGCAACTCC
HTR2C (*5-HT$_{2C}$R*) - human	NM_001256760.3	**F** CTAGTGGGACTACTTGTCATGCC
		**R** GCGATATAGCGCAGAGGTGCAT
GRM3 (*mGluR3*) - human	NM_000840.3	**F** GGGGCCTGTTTCCTATTAACG
		**R** TCACTCCTGGTAGCAAGTAATCA
Grm2 (*mGluR2*) - mouse	NM_001160353.1	**F** AGTGTGGACCTGTTAATGAGCA
		**R** CCAGGGCGTGTGTATCCTTG
Htr2a (*5-HT$_{2A}$R*) - mouse	NM_172812.3	**F** CCTGATGTCACTTGCCATAGCTG
		**R** CAGGTAAATCCAGACGGCACAG
Htr2c (*5-HT$_{2C}$R*) - mouse	NM_008312.4	**F** CTAATTGGCCTATTGGTTTGGCA
		**R** CGGGAATTGAAACAAGCGTCC
TAA-HA-mGluR2-TM	N/A	**F** GAATTCCTTTCCTAAATAAGTCCTTCTGTTGATCC
		**R** GGATCAACAGAAGGACTTATTTAGGAAAGGAATTC
TAA-HA-mGluR2-TAG-mCitrine	N/A	**F** CCCAAGCTGGCT AGCTAAGTCCTTCTGTTGATC
		**R** GATCAACAGAAGGACTTAGCTAGCCAG CTTGGG

N/A, not applicable; F, forward; R, reverse
