# Supplementary material for: Translation-independent association of mRNAs that encode protomers of the 5-HT2A-mGlu2 receptor complex
Source: J Biol Chem. 2025 Jun 26;301(8):110427. doi: 10.1016/j.jbc.2025.110427 (PMC12305237; doi:10.1016/j.jbc.2025.110427)
Supplement: Saha_supporting_tableS3 [file mmc5.pdf]

Supplementary Table S3: siRNA primer sequences

Gene Name	GenBank	siRNA sequence (5'-3')
HTR2A (*5-HT$_{2A}$R*) – human	NM_000839.5	CUAACACUUCUGAUGCAUU[dT][dT]
HTR2A (5-HT$_{2A}$R) – human	NM_000839.5	CUGAUAUAUGCUGCUGGGUUU[dT][dT]
GRM2 (*mGluR2*) – human	NM_000621.5	GAAUUCACUUGCGCUGAUU[dT][dT]
RPS24 – human	NM_033022.4	CCUGGAUUAUGCAAAGAAA[dT][dT]
RPS24 – human	NM_033022.4	CCCUGGAUUAUGCAAAGAA[dT][dT]
RPS5 – human	NM_001009.4	CGGUAUGCCGCCAAACGCU[dT][dT]

siRNA targeting *HTR2A* in row 1 was used in Figs. 1 and 2, whereas siRNA in row 2 was used in Fig. S1. siRNA targeting *RPS24* in row 4 was used in Fig. 9, while the one in row 5 was used in Fig. S7.
